# Associations between demographic, clinical and dietary factors and flares in inflammatory bowel disease: the PRognostic effect of Environmental factors in Crohn’s and Colitis (PREdiCCt) prospective cohort study

**DOI:** 10.1136/gutjnl-2025-337846

**Published:** 2026-01-19

**Authors:** Nathan Constantine-Cooke, Beatriz Gros, Nikolas Plevris, Linda J Williams, Gareth-Rhys Jones, Janet Kyle, Nicholas A Kennedy, Victor Velasco-Pardo, Alexander Rudge, Debbie Alexander, Carl A Anderson, Maiara Brusco de Freitas, Lisa M Derr, Lauranne AAP Derikx, Sian Gilchrist, Paul Henderson, Graham W Horgan, Peter Irving, Christopher A Lamb, Luke Jostins-Dean, James O Lindsay, Jonathan MacDonald, Craig Mowat, Charles Murray, Miles Parkes, Spyros I Siakavellas, Catalina A Vallejos, Daniel R Gaya, Jonathan M Rhodes, Alexandra M Johnstone, Christopher J Weir, Charlie W Lees, on behalf of the PREdiCCt study group, Suhail Ahmed

**Affiliations:** 1 Institute of Genetics and Cancer, University of Edinburgh Western General Hospital, Edinburgh, UK; 2 Gastroenterology Department, Reina Sofía University Hospital, IMIBIC, CIBERehd, Córdoba, Spain; 3 Edinburgh IBD Unit, Western General Hospital, Edinburgh, UK; 4 Usher Institute, The University of Edinburgh Usher Institute of Population Health Sciences and Informatics, Edinburgh, UK; 5 School for Infection and Immunity, University of Glasgow, Glasgow, UK; 6 Institute of Applied Health Sciences, University of Aberdeen, Aberdeen, UK; 7 Gastroenterology, Royal Devon and Exeter NHS Foundation Trust, Exeter, UK; 8 University of Exeter, Exeter, UK; 9 Wellcome Trust Genome Campus, Wellcome Trust Sanger Institute, Cambridge, UK; 10 Clinical Medicine, Aalborg Universitet, Copenhagen, Denmark; 11 The University of Edinburgh Usher Institute of Population Health Sciences and Informatics, Edinburgh, UK; 12 Department of Gastroenterology, Erasmus MC, Rotterdam, The Netherlands; 13 Department of Gastroenterology, NHS Fife, Kirkcaldy, UK; 14 Paediatric Gastroenterology and Nutrition, Royal Hospital for Children and Young People, Edinburgh, UK; 15 Child Life and Health, The University of Edinburgh Department of Child Life and Health, Edinburgh, UK; 16 Biomathematics and Statistics Scotland, Edinburgh, UK; 17 St Thomas’ Hospital, London, UK; 18 King’s College London, London, UK; 19 Translational & Clinical Research Institute, Newcastle University, Newcastle upon Tyne, UK; 20 Gastroenterology, Newcastle Upon Tyne Hospitals NHS Trust, Newcastle upon Tyne, UK; 21 Kennedy Institute of Rheumatology, Oxford University, Oxford, UK; 22 Barts and The London School of Medicine, Queen Mary University of London, London, UK; 23 Barts Health NHS Trust, London, UK; 24 NHS Glasgow and Greater Clyde, Glasgow, UK; 25 University of Dundee, Dundee, UK; 26 Gastroenterology, Royal Free Hospital, London, UK; 27 Department of Gastroenterology, Cambridge University Hospitals NHS Foundation Trust, Cambridge, UK; 28 Gastroenterology Department, University of Edinburgh Western General Hospital, Edinburgh, UK; 29 HDR UK, London, UK; 30 University of Liverpool Department of Molecular and Clinical Cancer Medicine, Liverpool, UK; 31 University of Aberdeen, The Rowett Institute, Aberdeen, UK; 32 The University of Edinburgh Usher Institute, Edinburgh, UK

**Keywords:** INFLAMMATORY BOWEL DISEASE, CROHN'S DISEASE, ULCERATIVE COLITIS, DIET

## Abstract

**Background:**

IBD is characterised by recurrent flares, but evidence on whether modifiable dietary factors influence flare risk is limited.

**Objective:**

The PREdiCCt study was designed to examine demographic, clinical and dietary factors associated with disease flare among patients with IBD in self-reported remission.

**Design:**

Multicentre, prospective cohort study conducted across 47 UK centres. Patients with Crohn’s disease (CD), ulcerative colitis (UC) or IBD unclassified (IBDU) in self-reported remission were prospectively followed up. The baseline diet was assessed using a validated food frequency questionnaire. The primary outcome was time to patient-reported flare (captured by monthly IBD-Control) and objective flare (clinical flare plus C-reactive protein >5 mg/L and/or faecal calprotectin (FC) >250 µg/g with treatment escalation). Associations were evaluated using Cox frailty models adjusted for demographic, clinical and biochemical variables, including baseline FC.

**Results:**

Between November 2016 and March 2020, 2629 participants (1370 CD; 1259 UC/IBDU) were enrolled and followed up for a median of 4.1 years (IQR 3.0–5.0). Baseline FC was strongly associated with patient-reported flares (FC ≥250 µg/g: adjusted HR (aHR) 2.22; FC 50–250 µg/g: aHR 1.52 (reference <50 µg/g)) and objective flares (FC ≥250 µg/g: aHR 3.25; FC 50–250 µg/g: aHR 1.98). In UC, higher total meat intake was associated with increased risk of objective flares (highest versus lowest quartile: aHR 1.95, 95% CI 1.07 to 3.56). No consistent associations were observed for ultraprocessed foods, fibre or polyunsaturated fatty acids and flare.

**Conclusion:**

Higher habitual meat intake was associated with increased risk of objective flare in UC, suggesting diet may contribute to flare susceptibility in specific patient groups.

**Trial registration number:**

NCT03282903.

**WHAT IS ALREADY KNOWN ON THIS TOPIC**

- Most prior studies on diet in IBD are cross-sectional or focus on exclusive enteral nutrition, with little prospective evidence on habitual diet and flare risk.

**WHAT THIS STUDY ADDS**

- This large prospective cohort study (the Prognostic Effect of Environmental Factors in Crohn’s and Colitis; n=2629) provides the most detailed evaluation to date of habitual diet and flare risk in IBD. Higher total meat intake was associated with an increased risk of objective flares in UC, independent of demographic, clinical and biochemical factors. No consistent associations were seen for ultraprocessed foods, fibre or polyunsaturated fatty acid intake.

**HOW THIS STUDY MIGHT AFFECT RESEARCH, PRACTICE OR POLICY**

- These findings highlight habitual diet as a potential modifiable risk factor for relapse in UC. Incorporating structured dietary assessment and counselling into routine care may support more preventive management strategies. The results also provide a foundation for designing targeted dietary intervention and mechanistic studies.

## Introduction

 The natural course of inflammatory bowel disease (IBD) is characterised by periods of remission and relapse,^1 2^ with uncontrolled inflammation contributing to disease progression. A key unanswered question in clinical management is what causes disease relapse and how we can anticipate impending disease flares. It is postulated that the same aetiopathogenic risk factors for IBD, including genetics,^3 4^ the environment,^5 6^ diet^7–9^ and the gut microbiome,^10^ are also likely to be involved in the disease course in individuals with established disease.

There is increasing evidence supporting the role of diet in IBD, as not only a risk factor for developing disease, but also a modifiable factor that influences inflammation in the gut.^11^ Epidemiological studies have shown individuals consuming diets high in vegetables and fish in the first year of life are at lower risk of developing IBD.^12^ Red meat consumption has been associated with the development of ulcerative colitis (UC),^13^ while high intake of ultraprocessed foods (UPFs) has been associated with the development of Crohn’s disease (CD).^14^ Similarly, animal models of colitis have also shown that diets high in plant-based fibres exert an anti-inflammatory effect, via alterations in the gut microbiota induced by production of short-chain fatty acids (SCFAs). Despite this, it is still unclear whether the same dietary patterns that predispose to the disease also contribute to an increased risk of flares with limited, high-quality data exploring dietary factors in the context of disease course and flare.

One of the most common questions patients will ask is, ‘What should I be eating?’. Recent European Crohn’s and Colitis Organisation guidelines recommend a Mediterranean diet for the majority of IBD patients, low red meat intake in UC and exclusive enteral nutrition to induce remission for CD.^15^ A better understanding of dietary factors can potentially lead to novel therapeutic approaches to both induce and maintain remission.

The Prognostic Effect of Environmental Factors in Crohn’s and Colitis (PREdiCCt) was designed as a prospective cohort study to help gain a better understanding of the factors implicated in disease flare in IBD. The primary objectives of PREdiCCt were to determine which aspects of (a) baseline habitual diet, (b) the environment, (c) genetic variation and (d) the gut microbiota are associated with disease flare in CD and/or UC and IBD unclassified (IBDU). Here, we present results from our first objective, specifically looking at habitual diet and disease flare.

## Methods

### Study design

PREdiCCt (https://www.clinicaltrials.gov/study/NCT03282903) is a large, multicentre, prospective cohort study. Adult and paediatric patients with a diagnosis of IBD were recruited across 47 sites within all four constituent countries of the UK from November 2016 to March 2020 (online supplemental table S1). Subjects were recruited from IBD clinics and were followed up for a minimum of 24 months to assess patient-reported flares (online supplemental figure S1). The full protocol and data analysis plan can be found online (https://www.constantine-cooke.com/predicct-analysis/).

### Participants

Inclusion criteria were: (1) confirmed diagnosis of CD or UC or IBDU as per Lennard-Jones and Porto criteria; (2) patient-reported clinical remission; (3) >6 months since diagnosis of IBD; (4) >2 months since any change in therapy for IBD; (5) ≥6 years of age at study entry. Exclusion criteria were: (1) patient not willing to take part in all aspects of study; (2) systemic corticosteroids (oral or intravenous) within the last 2 months; (3) immunomodulator (methotrexate, azathioprine, mercaptopurine) or advanced therapy (biologics, small molecules) commencement in the preceding 2 months; (4) unable to obtain written informed consent from patient or parent/guardian.

### Data collection

At study entry, several demographic and phenotypic details were recorded, along with routine laboratory markers collected as part of standard clinical practice (online supplemental table S2). The baseline disease phenotyping template was aligned to the UK IBD BioResource to facilitate co-recruitment and data sharing between studies.^16^ Bloods taken ±6 weeks of the date of recruitment were deemed valid if disease was in remission throughout this time and no change in therapy had occurred. Study participants were then asked to complete a series of baseline questionnaires looking at their environment, lifestyle and habitual diet, at home, via a custom-designed web portal (online supplemental table S3). In addition, participants provided a 4-day weighed food diary for diet microbiome analysis, sample of saliva (for genomic DNA) and two stool samples (for bacterial DNA, SCFA and faecal calprotectin (FC)) (*Genetic, microbiome and SCFA data will not be presented in this paper*). All patients were followed up prospectively for a minimum of 24 months. During the follow-up phase, participants were asked to complete a monthly update on their symptoms, treatment, environment and lifestyle through the web portal. More detailed online questionnaires were completed at 12 months and 24 months after enrolment, which were also designed to capture missing data from the prior monthly questionnaires. While follow-up via the web portal for patient-reported flares (see definitions below) was limited to 24 months, sites completed an end of study phenotyping form (median 43.7 months of follow-up; IQR 35.9–56.1), using local electronic medical records, to capture objective flares (see definitions below). These questionnaires were also designed to capture data that may have been missed from incomplete monthly questionnaires completed by patients online. No patient therapy/management was mandated/protocolised in the study.

### Flare data collection

Patient-reported flare was identified from the monthly online questionnaires completed by patients at home via the web portal. Patient-reported flare was defined as patients answering ‘*no*’ to the question ‘*Do you think your disease has been well controlled in the past 1 month?/Since you last logged into the portal?’* Patients who answered ‘no’ were prompted to provide an approximate date of when their disease was not well controlled. This was used to determine the date of flare. If a patient indicated being in flare, a stool sample testing kit for FC was posted out. The maximum follow-up for patient-reported flares was 24 months, collected prospectively via the web-portal questionnaires sent out to patients on a monthly basis.

Objective flare was defined as a patient-reported flare (defined above) plus commencement of any new medication (corticosteroids (intravenous hydrocortisone / methylprednisolone, prednisolone or budesonide), oral 5-aminosalicylic acid (5-ASA) (topical 5-ASA excluded), immunomodulator, advanced therapy) or dose escalation (increase in dose/frequency of 5-ASA, immunomodulator or advanced therapy) of existing medication to treat IBD flare, in addition to an increase in CRP (≥5 mg/L) and/or FC (≥250 µg/g). These data were collected via the end-of-study phenotyping forms completed for each patient by the recruiting site clinicians. The maximum follow-up for objective flares was the time from recruitment until the date the end-of-study phenotyping was completed for that subject. If a participant did not report a flare via the web portal, but their clinical care team identified worsening of symptoms during follow-up (eg, via IBD helpline, outpatient assessment, hospitalisation or surgery) via the end of study form, in addition to meeting the other criteria for an objective flare (see above), then they were included as having an objective flare at the date identified. If a flare was not reported by the participant despite their care team reporting an objective flare within 2 years, then a patient-reported flare was imputed at the time of objective flare.

Questionnaire responses over time are shown in online supplemental figure S2. As expected, there is a degree of attrition over time; however, there was a high response rate for the 24-month follow-up, which was designed to capture data missed on the monthly questionnaires. End of study phenotyping was completed for 94% (2476/2629) of participants within the study.

### Environmental, lifestyle and dietary data collection

All participants completed detailed environmental and lifestyle questionnaires within 7 days of their index visit, plus at 12 months and 24 months of follow-up. A list of all the environmental and lifestyle questionnaires is shown in online supplemental table S3. Baseline dietary data were collected using the Scottish Collaborative Group Food Frequency Questionnaire (SCG-FFQ V.6.6 (adults aged 18–64 years), C2 (for children aged 6–10 years) and C3 (for adolescents aged 11–17 years), www.foodfrequency.org) which is a previously validated semiquantitative 170 FFQ.^17^ Completed FFQs were reviewed for completeness at the University of Aberdeen and follow-up phone interviews were performed for any data queries.

### Faecal calprotectin analysis

A baseline FC was requested on the day of recruitment. Patients were given FC postal kits to complete at home. In addition, further FC postal kits were sent to patients if they indicated, via the monthly questionnaires, that their disease was not well controlled. Patients were advised to post samples on the same day as collection. All samples were posted directly to the biochemistry laboratories at the Western General Hospital, Edinburgh. On arrival at the laboratories, samples were stored at −20°C. FC was measured centrally for all samples using the standard ELISA technique (Calpro AS, Norway). FC values generated from samples collected as part of routine clinical care were also used to determine any objective flares that were missed and captured via the end of study phenotyping form. These samples were analysed at the site laboratory as per local protocols.

### Primary and secondary objectives

We looked to determine the association between baseline dietary factors and risk of disease flare in patients with IBD (CD, UC/IBDU) with self-reported clinical remission, controlling for mucosal inflammation. The primary objective was to test associations with the following dietary factors: (1) total animal protein intake; (2) dietary fibre; (3) polyunsaturated fatty acids (PUFAs) and (4) UPFs. The primary objectives shown here were revised from the original protocol, prior to the study completion, to include UPFs in replacement of emulsifiers and include PUFAs as a whole. This was because we were unable to accurately quantify emulsifiers from the FFQ data. Secondary objectives included associations between baseline demographic data and clinical data with patient-reported flare and objective flare. The primary outcome of interest was time to first patient-reported flare, with a secondary outcome of time to first objective flare.

### Study definitions

The overall PREdiCCt cohort was subdivided into two subcohorts: the FC cohort for those with baseline FC (n=2144) and the FFQ cohort for those with both baseline FC and FFQ data (n=1012) (table 1).

**Table 1:** Baseline description of the overall study cohort and subcohorts (FC cohort/FFQ cohort)

	Full cohort (N=2629)	FC cohort (n=2144)	FFQ cohort (n=1091)
Age, median (IQR)	44.0 (32.0–56.0)	44.0 (32.0–57.0)	47.0 (35.0–58.0)
Sex, Male	1207 (45.9%)	966 (45.1%)	476 (43.6%)
Ethnicity			
White	1862 (70.8%)	1657 (77.3%)	1039 (95.2%)
Non-white	71 (2.7%)	57 (2.7%)	24 (2.2%)
Missing	696 (26.5%)	430 (20.1%)	28 (2.6%)
BMI category			
Underweight >18.5 kg/m^2^	49 (1.9%)	38 (1.8%)	20 (1.8%)
Normal 18.5–24.9 kg/m^2^	1004 (38.2%)	840 (39.2%)	460 (42.2%)
Overweight 25–29.9 kg/m^2^	923 (35.1%)	751 (35.0%)	398 (36.5%)
Obese >30 kg/m^2^	552 (21.0%)	431 (20.1%)	191 (17.5%)
Missing	101 (3.8%)	84 (3.9%)	22 (2.0%)
IMD, median (IQR)	4 (2–5)	4 (2–5)	4 (3–5)
Missing	30 (1.1%)	22 (1.0%)	9 (0.8%)
Diagnosis			
CD	1370 (52.1%)	1118 (52.1%)	530 (48.6%)
UC	1174 (44.7%)	950 (44.3%)	523 (47.9%)
IBDU	85 (3.2%)	76 (3.5%)	38 (3.5%)
Montreal location*			
L1	362 (26.4%)	283 (25.3%)	149 (28.1%)
L2	333 (24.3%)	278 (24.9%)	141 (26.6%)
L3	454 (33.1%)	385 (34.4%)	160 (30.2%)
L4 only	14 (1.0%)	10 (0.9%)	6 (1.1%)
Missing	207 (15.1%)	162 (14.5%)	74 (14.0%)
Perianal disease at diagnosis*			
Yes	397 (29.0%)	321 (28.7%)	151 (28.5%)
No	746 (54.5%)	619 (55.4%)	299 (56.4%)
Missing	227 (16.6%)	178 (15.9%)	80 (15.1%)
Montreal behaviour*			
B1	749 (54.7%)	617 (55.2%)	285 (53.8%)
B2	252 (18.4%)	206 (18.4%)	105 (19.8%)
B3	121 (8.8%)	93 (8.3%)	54 (10.2%)
Missing	248 (18.1%)	202 (18.1%)	86 (16.2%)
Montreal extent†			
E1	166 (13.2%)	128 (12.5%)	81 (14.4%)
E2	503 (40.0%)	416 (40.5%)	250 (44.6%)
E3	318 (25.3%)	253 (24.7%)	130 (23.2%)
Missing	272 (21.6%)	229 (22.3%)	100 (17.8%)
IBD duration (years), median (IQR)	10.0 (4.73–18.6)	9.80 (4.60–18.6)	10.3 (4.92–20.1)
Smoking			
Current	122 (4.6%)	109 (5.1%)	63 (5.8%)
Ex-smoker	721 (27.4%)	627 (29.2%)	399 (36.6%)
Never	1071 (40.7%)	965 (45.0%)	597 (54.7%)
Missing	715 (27.2%)	443 (20.7%)	32 (2.9%)
Biologic exposure			
Current	920 (35.0%)	774 (36.1%)	310 (28.4%)
Previously	160 (6.1%)	133 (6.2%)	66 (6.0%)
Never prescribed	1549 (58.9%)	1237 (57.7%)	715 (65.5%)
IBD-Control-8			
Median (IQR)	13 (11–15)	13 (11–15)	13 (11–15)
Missing	718 (27.3%)	447 (20.8%)	38 (3.5%)
VAS <85	628 (23.9%)	569 (26.5%)	320 (29.3%)
HBI*, median (IQR)	2.00 (1.00–4.00)	2.00 (1.00–4.00)	2.00 (1.00–3.00)
Missing	833 (60.8%)	674 (60.3%)	287 (54.2%)
PMS†, median (IQR)	0 (0–1)	0 (0–1)	0 (0–1)
Missing	366 (29.1%)	291 (28.4%)	136 (24.2%)
FC (μg/g), median (IQR)	49.0 (20.0–161)	49.0 (20.0–161)	49.0 (20.0–161)
Missing	485 (18.4%)	0 (0%)	79 (7.2%)
CRP (mg/L), median (IQR)	2.00 (1.00–5.00)	2.00 (1.00–5.00)	2.00 (1.00–5.00)
Missing	637 (24.2%)	506 (23.6%)	280 (25.7%)
Surgery* Yes	634 (46.3%)	522 (46.7%)	273 (51.5%)
No	684 (49.9%)	561 (50.2%)	249 (47.0%)
Missing	52 (3.8%)	35 (3.1%)	8 (1.5%)

The FC cohort consists of subjects with a baseline FC measurement available. The FFQ cohort consists of subjects from the FC cohort who also completed a FFQ.

* CD patients only.

† UC/IBDU patients only.

BMI, Body Mass Index; CRP, C-Reactive-Protein; FC, faecal calprotectin; FFQ, Food Frequency Questionnaire; HBI, Harvey-Bradshaw Index; IBDU, IBD unclassified; IMD, Index of Multiple Deprivation; PMS, Partial Mayo Score; VAS, Visual Analogue Scale.

Patient-reported clinical remission for study entry was defined as patients answering ‘*yes*’ to a modified version of the first question in IBD-Control questionnaire: ‘*Do you think your disease has been well controlled in the past 1 month?*’. In CD, deep remission was defined as self-reported clinical remission in addition to Harvey-Bradshaw index ≤4 (adults) or weighted paediatric Crohn’s Disease Activity Index <12.5 (children), plus CRP <5 mg/L and FC <250 µg/g. In UC, deep remission was defined as self-reported clinical remission in addition to partial Mayo Score <2 (adults) or Paediatric Ulcerative Colitis Activity Index <10 (children), plus CRP <5 mg/L and FC <250 µg/g. Body Mass Index (BMI) categories were defined as per WHO guidelines. Index of Multiple Deprivation (IMD) for each subject has been calculated from subjects’ postcodes and government deprivation index databases held for each constituent nation of the UK. As was prespecified, IMD was not reweighted across the UK. This allowed all domains contributing to each nation’s IMD to be considered rather than discarding domains not shared across each IMD.

### Analysis of SCG-FFQ for dietary assessment

FFQ responses were converted into nutrient intakes using an in-house web-based data entry system. FFQ responses required no more than 10 missing data points to be considered sufficiently complete for analysis. The calculation package linked to the UK food databank with predefined portion sizes.^18^ Nutrients were expressed as a percentage of total energy intake. Dietary intakes were divided into quartiles across the distribution of the whole cohort, with the lowest assigned as the reference value. Dietary associations with flare were assessed by comparing quartiles, using the lowest quartile as the reference group. Unless otherwise specified, adjusted hazard ratio (aHR) compares the smallest and largest quartiles.

The NOVA score is commonly used to classify how processed food is, with NOVA 4 denoting UPF.^19^ However, this classification describes individual food items, not diet. As no gold standard exists for assessing the level of UPF in an individual’s diet, we used multiple approaches for categorising habitual UPF intake. An individual-specific score was calculated by summing multiplied standardised portion sizes and associated NOVA scores across food categories (online supplemental note 1). We also considered the percentage of calorie intake attributable to food classified as NOVA 4 in addition to exploring intake of food belonging to processed and unprocessed/minimally processed food groups. Across all dietary analyses, we controlled for diet quality index (as defined by Whybrow *et al*^20^) and BMI.

### Sample size

Prestudy sample size calculations assumed an annual flare rate of 15% with a dropout rate of 40% with initial sample size being 3100 individuals. If 1550 CD subjects and 1550 UC/IBDU subjects, the original target, were recruited, then this would provide 85% power to detect a HR of 0.6 when comparing across quartiles within either CD or UC/IBDU for dietary variables. However, the COVID-19 pandemic halted all recruitment, resulting in 1370 CD subjects and 1259 UC/IBDU subjects being recruited. Revised power calculations following the halt in recruitment, which assumed the same annual flare and dropout rates and based on 1018 completed FFQs gave 88.0% power to detect a HR of 0.6 (and 99% power to detect a HR of 0.5), using a two-sided 5% significance level. However, given significant dietary findings published in the literature that are typically found only within IBD types, we instead analysed CD and UC/IBDU separately. Power calculations indicated 60.1% power within each CD and UC/IBDU group to detect a HR of 0.6 and 87.0% to detect a HR of 0.5 when comparing dietary data quartiles.

### Statistical analysis

Descriptive statistics are presented as mean (SD) for normally distributed variables, and median (IQR) for non-normal variables. Categorical variables are presented as counts (percentages). χ^2^ and Fisher’s exact (for counts <5) tests were used to test for differences in proportions between subgroups. Primary exposure variables (intake of total animal protein, PUFA, dietary fibre and UPFs) were split into quartiles. Cox frailty regression models, which take into account variation in detectable flare rates across hospital sites, were used to examine the relationship between these variables and time to first patient-reported and objective flare. For each exposure variable of interest, potential prespecified confounders measured at baseline (sex, IMD, FC (categorical:<50 µg/g, 50–250 µg/g, >250 µg/g)) were adjusted for as fixed effects. FC was treated as a categorical variable using these cut-offs as they are clinically relevant and broadly reflect inactive, minimally active and active mucosal inflammation, respectively. Further analysis assessing FC as a continuous variable was also performed (online supplemental figure S3). Although smoking status was originally planned to be controlled for, the high degree of missingness for these data (n=715 not available for overall cohort) and the lack of significant associations with time to flare (online supplemental figure S4) resulted in smoking status not being controlled for in these analyses. However, as a sensitivity analysis, missing smoking data were imputed via multiple imputation by chained equations, with analyses of prespecified dietary variables re-ran (online supplemental figures S5–S8).

For key significant findings, population-averaged 2-year absolute risk of flare was calculated from Cox models, stratifying by the variable of interest.^21^

For dietary variables, diet quality index and BMI were also controlled for. Results are expressed as aHRs with 95% CIs. Model diagnostics were explored via a test based on Schoenfeld residuals to assess the proportional hazards assumption,^22^ measuring the difference in each parameter estimate with and without individual data points to assess influential outliers, and inspecting Martingale residuals which allow the functional form of covariates and the proportional hazards assumption to be assessed.^23^ Analyses were repeated for both the primary and secondary outcomes of patient-reported flare and objective flare, respectively.

Missingness patterns were visualised using the finalfit R package (https://finalfit.org/). As missingness for variables being controlled for was low, complete case analyses were performed. While loss of follow-up may have impacted capture of patient-reported flares, it is less of a concern for objective flares due to the opportunity for flares of this type to be captured via end-of-study phenotyping which was completed for 94% of the cohort.

To further support the robustness of our univariate analyses, we compiled unadjusted and Bonferroni adjusted p values for all tested associations not specified as primary variables in the statistical analysis plan. These results are presented in online supplemental table S7.1–S7.6.

Statistical analysis was performed using R (V.4.4.0). Supplemental statistical reports describing data processing and all analyses are available online (https://www.constantine-cooke.com/predicct-analysis/). A Docker image and all R analysis code are also publicly available (https://github.com/nathansam/predicct-analysis).

### Patient and public involvement

Multiple patient focus groups were conducted throughout the design and recruitment phase of PREdiCCt. Participants and interested members of the public were kept informed and updated through regular email newsletter updates and via social media channels. The PREdiCCt trial steering committee included two patient representatives for the duration of the study.

## Results

### Study population

**Figure 1:**
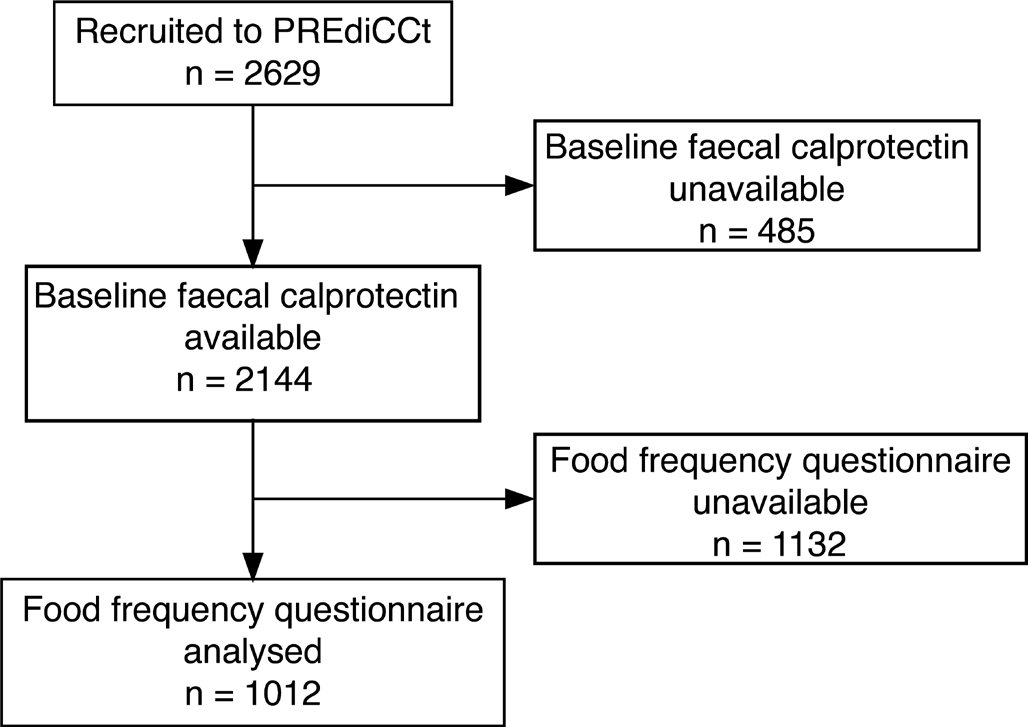
PRISMA diagram showing the derivation of the food frequency questionnaire cohort.

A total of 2629 patients with IBD in self-reported clinical remission were recruited across 47 sites in the UK from November 2016 to March 2020 (figure 1, table 1). Of these, 1370 (52.1%) had CD and 1259 (47.9%) had UC/IBDU (table 1). The median age of the cohort was 44 years (IQR 32–56) and consisted of 1422 females (54.1%). Median disease duration at recruitment was 10.0 years (IQR 4.7–18.6) (online supplemental figure S9). Ethnicity of the cohort was 70.8% white (26.5% missing, 1862/2629). Across the cohort, 55.1% of patients were overweight or obese (BMI ≥25 kg/m^2^, 1475/2629), comparable to the general UK population (table 1). Patients were prospectively followed up for a median duration of 4.1 years (IQR 3.0–5.0). Seasonality of patient recruitment was consistent throughout the year (online supplemental figure S10).

Baseline characteristics between cohorts (overall, FC and FC plus FFQ) were equivalent, other than subjects in the FFQ cohort being older (median age: 47 years vs 44 years in both the FC and overall cohorts, respectively) and having lower rates of current biologic use at recruitment (28.4% in the FFQ cohort vs 36.1% and 35% in FC and overall cohorts, respectively) (table 1, online supplemental table S4).

### Faecal calprotectin cohort

A total of 2144 patients were included in the FC cohort (table 2). The median IBD-Control 8 score was 13 (IQR 11–15), with 52.3% (n=1121) of the cohort having an IBD-Control Visual Analogue Scale (VAS) score of >85 (table 1). Baseline median CRP and FC of the cohort was 2.0 mg/L (1.0–5.0 mg/L) and 49.0 µg/g (IQR 20.0–161 µg/g)**,** respectively (table 1). A total of 1077 (52.3%), 683 (31.4%) and 384 (15.3%) patients had a FC <50 µg/g, between 50 µg/g and 250 µg/g, and ≥250 µg/g, respectively (table 2). While we did observe some subtle differences in the proportion of patients with CD versus UC/IBDU across three FC categories (more patients in the UC/IBDU group had a FC <50 µg/g), the groups were broadly similar (table 2). There were no differences observed in IBD phenotype at recruitment between the three FC categories in either CD or UC/IBDU (online supplemental table S5.1 and S5.2).

**Table 2:** Baseline phenotyping of FC cohort (n=2144) stratified by FC cut-off value (<50 µg/g, 50–250 µg/g and ≥250 µg/g)

FC cohort	FC <50 µg/g (n=1077)	FC 50–250 µg/g (n=683)	FC >250 µg/g (n=384)	P value
Age, median (IQR)	44.0 (33.0–56.0)	46.0 (33.0–59.5)	42.5 (30.0–55.0)	**0.004**
Sex				**< 0.001**
Male	442 (41.0%)	333 (48.8%)	191 (49.7%)	
Female	635 (59.0%)	350 (51.2%)	193 (50.3%)	
Index of Multiple Deprivation	4 (3–5)	4 (2–5)	4 (2–5)	0.39
Missing	*15* (*1.4%*)	*< 5*	*< 5*	
Diagnosis				**0.03**
CD	533 (49.5%)	381 (55.8%)	204 (53.1%)	
UC	504 (46.8%)	283 (41.4%)	163 (42.4%)	
IBDU	40 (3.7%)	19 (2.8%)	17 (4.4%)	
IBD duration, median (IQR)	10.1 (4.62–19.4)	9.66 (4.76–18.4)	9.07 (4.42–16.5)	0.06
Missing	*60* (*5.6%*)	*38* (*5.6%*)	*22* (*5.7%*)	
Smoking				0.07
Current	64 (5.9%)	36 (5.3%)	9 (2.3%)	
Ex-smoker	309 (28.7%)	213 (31.2%)	105 (27.3%)	
Never	487 (45.2%)	300 (43.9%)	178 (46.4%)	
Missing	*217* (*20.1%*)	*134* (*19.6%*)	*92* (*24.0%*)	
Biologic exposure				**0.002**
Current	406 (37.7%)	220 (32.2%)	148 (38.5%)	
Previously	48 (4.5%)	55 (8.1%)	30 (7.8%)	
Never prescribed	623 (57.8%)	408 (59.7%)	206 (53.6%)	
Surgery				**0.025**
Yes	251 (47.1%)	193 (50.7%)	78 (38.2%)	
No	262 (49.2%)	181 (47.5%)	118 (57.8%)	
Missing	*20* (*3.8%*)	*7* (*1.8%*)	*8* (*3.9%*)	
Haemoglobin, median (IQR)	139 (130–147)	139 (130–148)	137 (127–146)	0.08
Missing	*184* (*17.1%*)	*120* (*17.6%*)	*59* (*15.4%*)	
CRP, median (IQR)	2.00 (1.00–5.00)	2.00 (1.00–5.00)	4.00 (1.00–6.00)	**< 0.001**
Missing	*256* (*23.8%*)	*167* (*24.5%*)	*83* (*21.6%*)	
Albumin, median (IQR)	42.0 (39.0–46.0)	41.0 (38.0–44.0)	40.0 (38.0–43.0)	**< 0.001**
Missing	*226* (*21.0%*)	*146* (*21.4%*)	*76* (*19.8%*)	
FC, median (IQR)	20.0 (20.0–29.0)	104 (71.0–157)	498 (347–780)	–
IBD-Control-8, median (IQR)	13.0 (11.0–15.0)	13.0 (11.0–15.0)	13.0 (9.00–15.0)	**0.04**
Missing	*218* (*20.2%*)	*136* (*19.9%*)	*93* (*24.2%*)	
IBD-Control-8 - VAS				**< 0.001**
<85	250 (23.2%)	201 (29.4%)	118 (30.7%)	
85+	607 (56.4%)	342 (50.1%)	172 (44.8%)	
Missing	*220* (*20.4%*)	*140* (*20.5%*)	*94* (*24.5%*)	

Boldface font indicates significant at the 5% level.

CRP, C-Reactive Protein; FC, faecal calprotectin; IBDU, IBD unclassified; VAS, Visual Analogue Scale.

### FFQ cohort

Sufficiently complete dietary data comprising macronutrient profiles from FFQs were available for 1091 participants, of whom 1091 (41.5%) also had FC available (n=530 (48.6%) with CD, n=561 (51.4%) with UC/IBDU) (table 3). In this subset of patients, median BMI was 25.5 (IQR: 22.8–28.6) and daily calorie intake was 2219 kcal (1810–2731) (table 1). This predominantly originated from carbohydrates (47.6%) and fat (35.6%) (including saturated fatty acids (13.3%), monounsaturated fatty acids (12.6%) and PUFA (2.5%)) (table 3, online supplemental figure S11). Median protein intake was 91.7 g/day (16.5% of calorie intake), of which a median of 36.1 g/day was from meat and fish sources, 18.3 g/day from dairy, 0.9 g/day from eggs and 33.5 g/day from plant-based protein sources (table 3, online supplemental figure S11). A total of 40.2% of the total energy intake was attributable to UPFs (NOVA score of 4) (table 3, online supplemental figure S11). The median diet quality index was 34.0 (IQR 26.6–41.4) (online supplemental figure S12). There were baseline differences in calorie intake between UC and CD patients, with higher daily calorie intake in UC versus CD (median 2269 vs 2160, p=0.033) (table 3).

**Table 3:** Dietary data of FFQ cohort separated by diagnosis type and sex

	UC/IBDU			CD			Female P value	Male P value	CD/UC P value
	Female	Male (n=239)	P value	Female	Male (n=205)	P value			
FC	49 (20–156)	51 (20–157)	0.913	46 (20–160)	45 (20–183)	0.885	0.933	0.826	
Energy (kcal/day)	2264 (1797–2860)	2281 (1900– 2749)	0.828	2165 (1811–2628)	2155 (1760–2729)	0.885	0.054	0.341	**0.033**
Fat (g/day)	91 (72–117)	89 (72–112)	0.755	89 (70–109)	90 (68–114)	0.687	0.557	0.703	0.801
Saturated fat (g/day)	34 (26–45)	34 (26–42)	0.967	33 (25–43)	33 (25-43)	0.885	0.557	0.849	0.529
Carbohydrates (g/day)	268 (200–335)	272 (216–318)	0.694	251 (208–313)	260 (202–319)	0.443	0.154	0.253	**0.044**
Protein (g/day)	95 (74–116)	94 (76–110)	0.755	91 (72–115)	89 (71–111)	0.610	0.275	0.128	0.078
Ultraprocessed food %	39.6 (32.2–48.4)	40.3 (30.8–49.1)	0.828	40.4 (32.2–47.9)	40.4 (32.4–48.8)	0.970	0.801	0.999	0.801
Fat %	35.8 (33.0–38.8)	34.8 (31.7–38.4)	0.506	35.6 (32.7–38.6)	35.5 (32.7–38.4)	0.970	0.933	0.446	0.801
Saturated fat %	13.4 (11.6–15.0)	12.8 (11.4–14.6)	0.967	13.3 (11.5–14.9)	13.5 (11.7–14.8)	0.885	0.801	0.057	0.258
Carbohydrates %	47.5 (43.6–51.5)	47.8 (43–52.4)	0.694	47.6 (43.7–51.6)	47.2 (43.6–51.2)	0.610	0.933	0.703	0.900
Alcohol (g/d)	6.7 (0.1–15.3)	7.4 (1.4–17.6)	0.569	7.0 (1.9–15.1)	7.3 (2.4–14.9)	0.970	0.933	0.918	0.900
Alcohol %	2.1 (0.02–4.5)	2.2 (0.4–4.7)	0.569	2.2 (0.6–4.4)	2.3 (0.8–4.6)	0.826	0.557	0.568	0.529
Alcohol (U/day)	0.8 (0.01–1.9)	0.9 (0.2–2.2)	0.569	0.9 (0.2–1.9)	0.9 (0.3–1.8)	0.970	0.933	0.918	0.900
Fibre NSP (g/day)	17.7 (12.8–22.6)	17.4 (13.2–21.9)	0.892	16.4 (12.7–21.7)	17.2 (12.3–21.9)	0.558	0.208	0.849	0.314
Sugars (g/day)	113.5 (85.1–154.2)	118.3 (92.9–152.1)	0.202	109.4 (83.6–142.9)	113.1 (86.7–149.3)	0.342	0.675	0.446	0.131
Calcium (mg/day)	1192.3 (859.1–1492.8)	1094.8 (850.1–1411.6)	0.122	1072.6 (820.5–1379.4)	1118.1 (856.7–1386.9)	0.443	**0.036**	0.703	0.209
Magnesium (mg/day)	334.4 (264.1–424.0)	346.1 (271.9–410.7)	0.356	324.7 (269.3–408.9)	335.6 (266.3–412.3)	0.342	0.402	0.446	0.167
Iron (mg/day)	13 (10.5–17.1)	13.5 (10.6–16.3)	0.356	13.2 (10.5–15.7)	12.9 (9.9–15.9)	0.382	0.801	0.128	0.314
Zinc (mg/day)	11.2 (8.8–14.2)	11.0 (8.8–13.6)	0.892	10.8 (8.5–13.4)	10.4 (8.5–13.1)	0.290	0.557	0.183	0.131
Potassium (mg/day)	3657.4 (2875.5–4592.9)	3851.7 (3194.3–4444.6)	0.071	3588.9 (2900.3–4387.5)	3657.2 (2856.3–4589.4)	0.826	0.557	0.128	0.131
Thiamin (mg/day)	1.7 (1.4–2.2)	1.8 (1.4–2.1)	0.202	1.7 (1.4–2.0)	1.7 (1.3–2.1)	0.744	0.801	**0.013**	0.102
Riboflavin (mg/day)	2.1 (1.6–2.8)	2.0 (1.6–2.6)	0.232	1.9 (1.5–2.6)	2.0 (1.5–2.5)	0.305	0.054	0.999	0.131
Niacin (mg/day)	20.3 (16.4–26.3)	21.3 (16.8–26.2)	0.202	20.5 (16.3–25.6)	20.5 (16.1–25.4)	0.970	0.801	0.183	0.529
Vit B6 (mg/day)	1.8 (1.4–2.3)	1.9 (1.5–2.3)	**0.048**	1.7 (1.4–2.2)	1.8 (1.4–2.2)	0.342	0.111	0.057	**0.023**
Vit B12 (ug/day)	7.0 (4.8–10.3)	7.3 (5.1–10.4)	0.569	7.4 (5.1–10.5)	7.2 (4.9–10.1)	0.610	0.557	0.703	0.801
Folic acid (ug/day)	262.3 (191.1–349.1)	266.7 (202.7–329.4)	0.967	247.1 (201.8–318.8)	255.9 (197.3–317.1)	0.443	0.154	0.849	0.167
Vit C (mg/day)	94.1 (62.4–139.9)	101.8 (69.3–146.0)	0.272	99.4 (63.5–142.5)	107.7 (66.6–148.6)	0.257	0.356	0.446	0.258
Vit E (mg/day)	10.4 (7.9–13.7)	10.3 (8.2–13.7)	0.967	9.8 (8.0–12.9)	10.3 (7.7–13.3)	0.443	0.275	0.999	0.529
Vit D (ug/day)	3.6 (2.3–5.8)	3.9 (2.7–5.7)	0.569	3.9 (2.4–5.7)	3.7 (2.3–6.1)	0.610	0.557	0.849	0.801

Boldface font indicates significant at the 5% level.

CD, Crohn’s disease; FC, faecal calprotectin; FFQ, Food Frequency Questionnaire; IBDU, IBD unclassified.

Analysis of dietary components based on baseline inflammatory activity revealed that in UC, lower baseline FC was associated with higher PUFA intake (p=0.03), higher diet quality index (p=0.03), lower processed meat intake (p=0.002) and higher vegetable/legume intake (p=0.02). No dietary component associations were seen with baseline FC values in patients with CD.

### Flare occurrence

**Figure 2:**
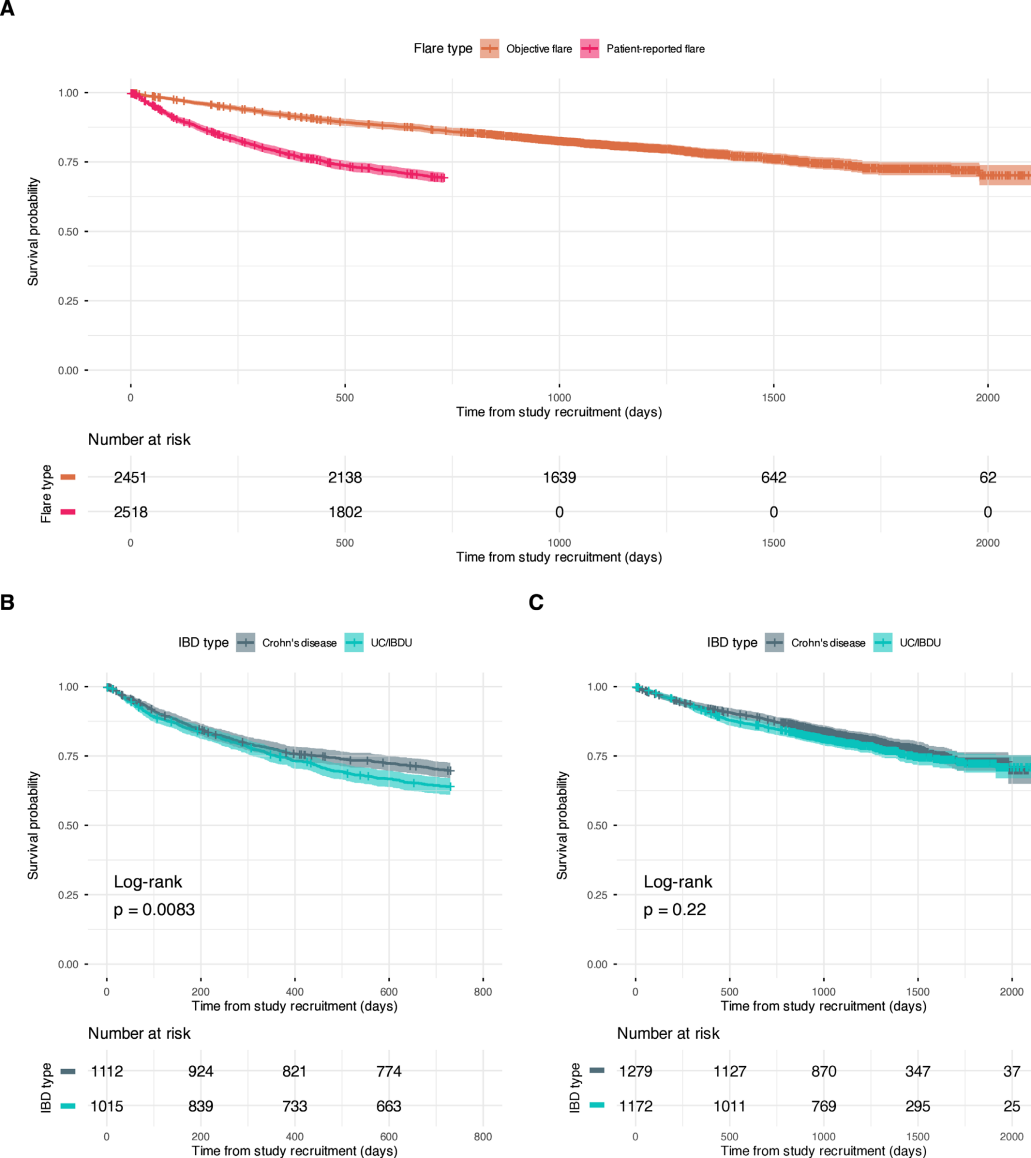
(A) Overall patient-reported and objective flares, (B) Patient-reported flares stratified by diagnosis (CD vs UC/IBDU), (C) Objective flares stratified by diagnosis (CD vs UC/IBDU). A log-rank test is not performed in (A) as objective flares require a patient-reported flare and the events are therefore not mutually exclusive. IBDU, IBD unclassified.

During the initial 24-month follow-up period, the cumulative patient-reported flare rate was 31% (95% CI 29% to 32%), while the cumulative objective flare rate was 14% (95% CI 12% to 15%) (figure 2A). Objective flares accumulated at a mean of 5.6% per year over 4 years of follow-up (figure 2A). At 24 months, cumulative patient-reported and objective flare rates were 28% and 12% in CD, and 33% and 15% in UC/IBDU, respectively (figure 2B,C). A greater proportion of patients with UC/IBDU had a patient-reported flare compared with patients with CD (log-rank p=0.018) (figure 2B). No difference was observed in objective flare rates between CD and UC/IBDU (p=0.075) (figure 2C). After extended follow-up (median 3.1, IQR 2.4–4.1 years), the cumulative objective flare rate increased to 30% (95% CI 26% to 33%) (figure 2A). Again, no difference was observed in objective flare rates between CD and UC/IBDU at the end of extended follow-up (p=0.22) (figure 2C).

### Primary analysis of phenotypic, clinical and biochemical variables associated with flare occurrence

**Figure 3:**
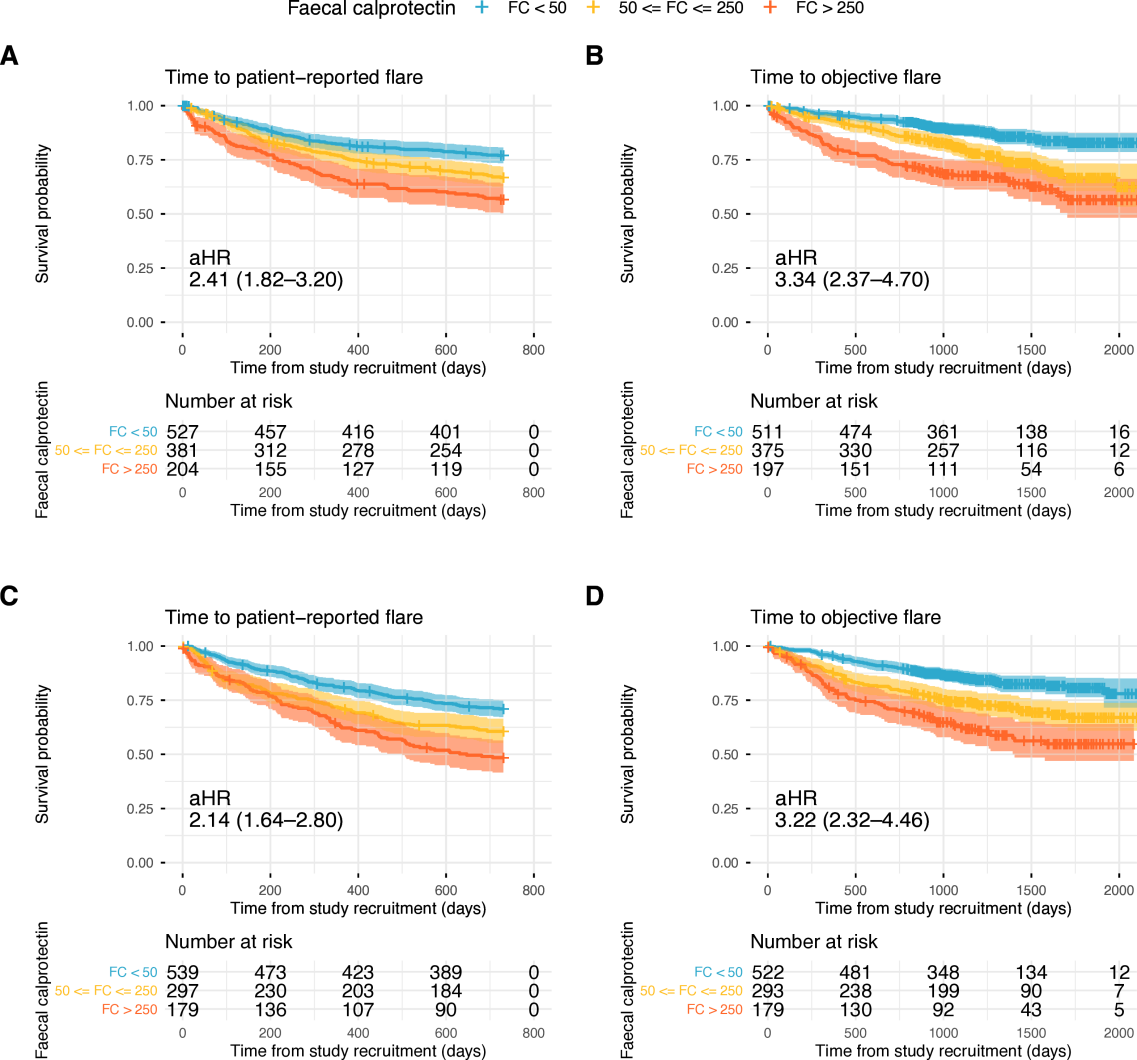
Flares by faecal calprotectin (FC) stratified into FC < 50, 50 ≤ FC ≤ 250, and FC >250 μg/g. (A) Patient-reported flare in Crohn’s disease; (B) objective flare in Crohn’s disease; (C) patient-reported flare in ulcerative colitis/inflammatory bowel disease unclassified; (D) objective flare in ulcerative colitis/inflammatory bowel disease unclassified. aHR, adjusted hazard ratio.

Of the prespecified variables, we controlled for sex, IMD and FC, and multivariable Cox regression analysis revealed female sex was associated with patient-reported flare in patients with both CD (aHR 2.00, 95% CI 1.58 to 2.56) and UC/IBDU (aHR 1.54, 95% CI 1.25 to 1.91) (table 4A,C, online supplemental figure S13). In CD, a FC value of 50–250 µg/g and >250 µg/g was associated with an increased risk of both patient-reported (aHR 1.58, 95% CI 1.23 to 2.05 and aHR 2.41, 95% CI 1.82 to 3.20, respectively) and objective flare (aHR 2.02, 95% CI 1.47 to 2.78 and aHR 3.34, 95% CI 2.37 to 4.70, respectively) when compared with a value of <50 µg/g (figure 3). This equated to an 8% chance of an objective flare within 2 years for FC <50 µg/g and a 26% chance of a objective flare occurring in the same time period for FC >250 µg/g. Similarly, in UC, an FC value of 50–250 µg/g and >250 µg/g was associated with an increased risk of both patient-reported (aHR 1.57, 95% CI 1.23 to 2.01 and aHR 2.15, 95% CI 1.64 to 2.80, respectively) and objective flare (aHR 2.03, 95% CI 1.49 to 2.77 and aHR 3.22, 95% CI 2.33 to 4.46, respectively) when compared with a value of <50 µg/g (table 4C,D, figure 3). The absolute risk of an objective flare occurring within 2 years was 11% for FC <50 µg/g and 34% for FC >250 µg/g. Area-based IMD was not associated with time to first patient-reported or objective flare in either CD or UC/IBDU (online supplemental figure S14). A sensitivity analysis, assessing FC as a continuous variable in the model, demonstrated similar results (online supplemental figure S3). A sensitivity analysis was performed including baseline medication in the model that again demonstrated similar results (https://www.constantine-cooke.com/predicct-analysis/Survival/Diet.html).

**Table 4:** Multivariable Cox regression analysis for prespecified baseline variables (sex, IMD, FC category) and risk of (A) patient-reported and (B) objective flare in CD; (C) patient-reported flare and (D) objective flare in UC/IBDU.

(A)				
CD: patient-reported flare	aHR	Lower 95%	Upper 95%	P value
Female sex	2.00	1.58	2.54	**<0.001**
IMD2	0.94	0.60	1.47	0.775
IMD3	0.89	0.56	1.40	0.607
IMD4	0.94	0.61	1.46	0.789
IMD5	0.99	0.64	1.51	0.947
FC 50–250 (μg/g)	1.58	1.23	2.04	**<0.001**
FC >250 (μg/g)	2.41	1.82	3.20	**<0.001**

(B)				
CD: objective flare	aHR	Lower 95%	Upper 95%	P value
Female sex	1.39	1.06	1.82	**0.018**
IMD2	0.92	0.54	1.58	0.77
IMD3	0.97	0.56	1.68	0.91
IMD4	0.90	0.52	1.53	0.69
IMD5	0.90	0.54	1.52	0.70
FC 50–250 (μg/g)	2.02	1.47	2.78	**<0.001**
FC >250 (μg/g)	3.34	2.37	4.70	**<0.001**

(C)				
UC: patient-reported flare	aHR	Lower 95%	Upper 95%	P value
Female sex	1.54	1.25	1.91	**<0.001**
IMD2	1.24	0.79	1.97	0.35
IMD3	1.10	0.70	1.73	0.68
IMD4	1.44	0.94	2.22	0.10
IMD5	1.20	0.79	1.83	0.40
FC 50–250 (μg/g)	1.57	1.23	2.01	**<0.001**
FC >250 (μg/g)	2.14	1.64	2.80	**<0.001**

(D)				
UC: objective flare	aHR	Lower 95%	Upper 95%	P value
Female sex	1.33	1.02	1.72	**0.035**
IMD2	1.41	0.79	2.53	0.25
IMD3	1.38	0.78	2.42	0.27
IMD4	1.75	1.01	3.02	**0.045**
IMD5	1.30	0.76	2.23	0.34
FC 50–250 (μg/g)	2.03	1.49	2.77	**<0.001**
FC >250 (μg/g)	3.22	2.32	4.46	**<0.001**

Boldface font indicates significant at the 5% level.

aHR, adjusted hazard ratio; CD, Crohn's disease; FC, faecal calprotectin; IMD, Index of Multiple Deprivation.

**Figure 4:**
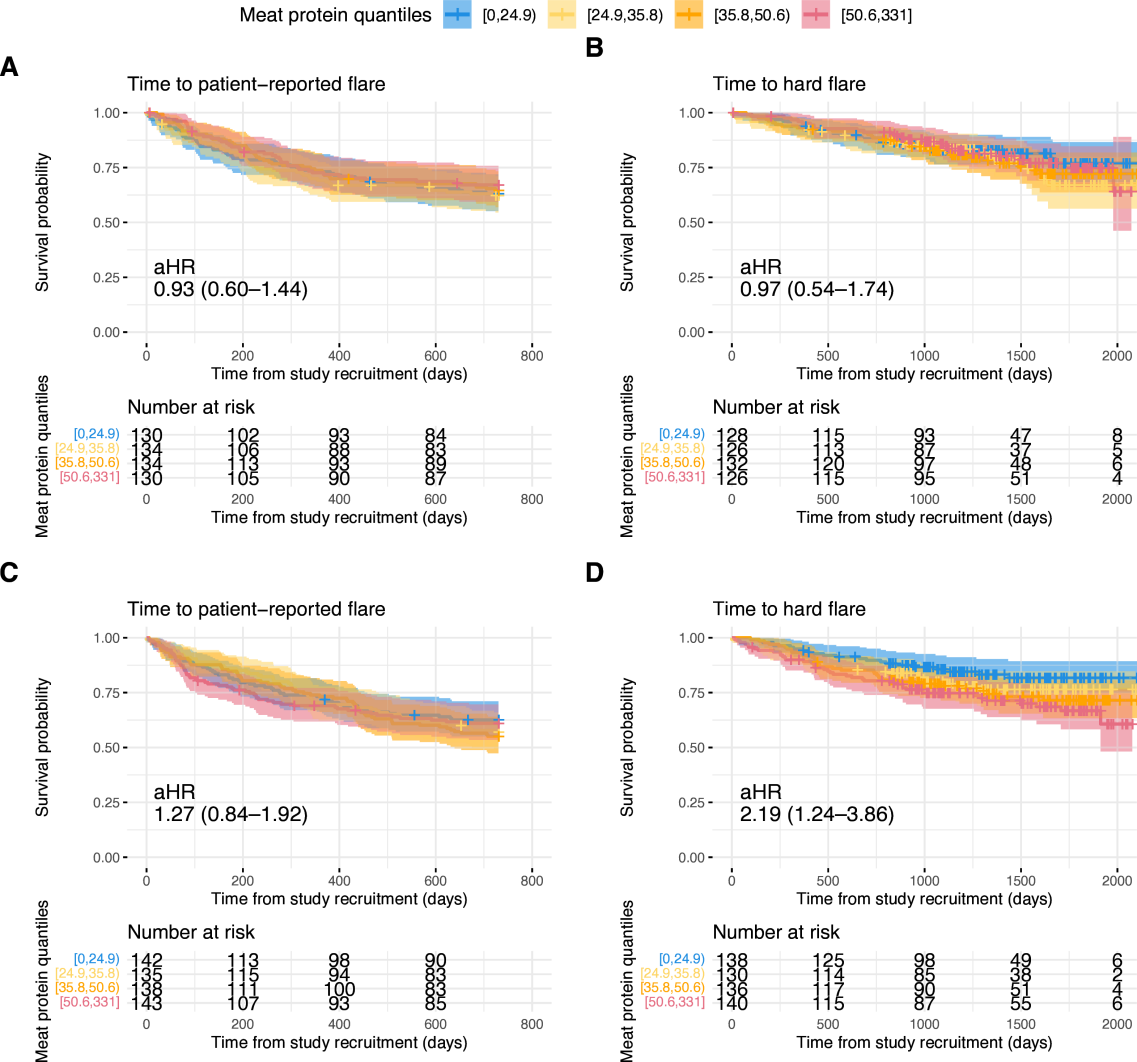
Flares stratified by quartiles of protein intake from meat sources describing (A) patient-reported flares in Crohn’s disease; (B) objective flares in Crohn’s disease; (C) patient-reported flares in ulcerative colitis; and (D) objective flares in ulcerative colitis. aHR, adjusted hazard ratio.

### Primary analysis of associations between dietary composition and flare occurrence

Association between baseline dietary constituents, stratified into quartiles, and patient-reported as well as objective flare in CD and UC/IBDU was analysed (adjusted for the prespecified variables as per section 3.5). In UC/IBDU, increased meat (white/red and fish) consumption was associated with an increased risk of objective flare (aHR 2.19; 95% CI 1.24 to 3.86) (figure 4D). Those in the lowest quartile had a 12% chance of an objective flare occurring within 2 years, while those in the top quartile had a 26% chance of flaring in the same time period. In addition, overall meat intake (aHR 1.95, 95% CI 1.07 to 3.56), unprocessed red meat (aHR 1.81, 95% CI 1.15 to 2.84) and unprocessed white meat (aHR 1.91, 95% CI 1.10 to 3.30), but not fish (aHR 1.16; 95% CI 0.70 to 1.94), was associated with objective flare in UC (online supplemental figure S15). This finding remained consistent when modelling overall meat intake as a continuous variable (online supplemental figure S16). No association was found for patient-reported flare and baseline meat intake in UC, nor for patient-reported or objective flare in CD (figure 4A–C).

Fibre and PUFA intake was not associated with risk of patient-reported or objective flare in either CD or UC/IBDU (online supplemental figures S17 and S18). UPFs, when considered as a percentage of calorie intake at baseline, were associated with a lower risk of patient-reported flare in patients with UC (aHR 0.59, 95% CI 0.39 to 0.88), but no other associations were observed across UPF analyses in UC or CD (online supplemental figure S19). Alcohol intake was not associated with risk of patient-reported or objective flare in either CD or UC/IBDU. A detailed breakdown of all dietary analyses is available online (https://www.constantine-cooke.com/predicct-analysis/Survival/Diet.html). A sensitivity analysis was also performed including baseline medication in the model that again demonstrated similar dietary results (online only).

### Phenotypic, clinical and biochemical variables associated with flare occurrence

Additional multivariable Cox regression analysis adjusting for the prespecified variables (as per section 3.5) revealed that older age was associated with a reduced risk of objective flare in CD (aHR 0.99, 95% CI 0.98 to 1.00) and UC (aHR 0.99, 95% CI 0.98 to 1.00). In patients with CD, upper GI disease involvement (+L4) (aHR 1.57, 95% CI 1.07 to 2.30) and previous surgery (aHR 0.75, 95% CI 0.57 to 0.98) were associated with objective flare. In patients with UC, left side colitis (E2) (aHR 2.15, 95% CI 1.26 to 3.66) and extensive colitis (E3) (aHR 1.85, 95% CI 1.05 to 3.25) had a greater risk of objective flare when compared with those with isolated proctitis (E1).

IBD-Control-8 questionnaire and VAS at baseline showed that higher IBD-Control-8 was associated with fewer patient-reported flares in CD and UC (aHR 0.89, 95% CI 0.86 to 0.92 and aHR 0.92, 95% CI 0.89 to 0.96 respectively), but only to objective flare in UC (aHR 0.95, 95% CI 0.90 to 0.99). Similar findings were observed with VAS, with a VAS score >85 being associated with patient-reported flare in both CD and UC (aHR 0.57, 95% CI 0.45 to 0.73 and aHR 0.69, 95% CI 0.54 to 0.87) but only associated with objective flare in UC (aHR 0.66, 95% CI 0.48 to 0.89). From biochemical parameters, haemoglobin levels and white cell count were associated with patient-reported flare in UC (aHR 0.99, 95% CI 0.98 to 1.00 and aHR 1.08, 95% CI 1.02 to 1.15, respectively) but not associated with objective flare. Baseline CRP was not associated with patient-reported flare nor objective flare in either CD or UC. However, there is a strong association between elevated FC and CRP (online supplemental figure S20), likely precluding associations with flare.

## Discussion

The PREdiCCt study is the largest prospective trial to date designed to elucidate clinical, environmental and lifestyle factors associated with disease flares in IBD. Among patients in self-reported clinical remission, we identified that higher baseline meat consumption, both red and white, was associated with an increased risk of objective flares in patients with UC, but not in CD. While meat intake was associated with baseline FC level in UC patients, a strength of the present study is that FC (along with sex and social deprivation) was controlled for in the survival analysis, acting as a robust proxy for residual mucosal inflammation. This association supports previous findings linking red and processed meat to an increased risk of developing UC^24^ and colorectal cancer.^25^ Mechanistically, this may relate to the generation of proinflammatory and carcinogenic compounds such as haem iron, nitrates/nitrites and thermally generated heterocyclic amines that can impair barrier integrity, promote oxidative stress and skew the colonic microbiome toward proinflammatory taxa.^26^ Recent evidence from the IBD Partners study supports this, showing that patients in the highest quartile of red meat consumption had over twice the odds of UC flare.^27^

In contrast, we did not observe an association between fibre or PUFA intake and flare risk in either IBD subtype. Although earlier studies suggested a protective effect of high fibre intake in CD (adjusted OR for the highest versus lowest quartile: 0.58)^28^ and potential benefit from low PUFA diets such as the Mediterranean diet,^29^ the precise role of these dietary constituents remains unclear. For example, it is increasingly recognised that not all types of fibre are similar in their capacity to modulate the gut microbiota and support mucosal immunity via mechanisms such as SCFA production.^30^ Of note, in our study, we could not differentiate soluble from insoluble fibre as there is no database for dietary assessment of these fibres separately.

Interestingly, higher intake of UPFs was associated with reduced risk of patient-reported flares in UC, although no effect was observed for objective flares, and higher intake of fish was associated with increased patient-reported flares. These counterintuitive results may reflect residual confounding, such as reverse causality, unmeasured diet quality variables or a misclassification of functional symptoms in the absence of objective inflammation. Existing evidence primarily implicates UPFs in IBD incidence rather than flare risk, and prior studies reporting increased flare risk related to UPF in IBD were limited in size and scope. It should be noted that the average UPF consumption within the cohort was lower compared with data describing the general UK population, which predicted 55% of calorie intake as being obtained from UPFs.^31^ This potentially has implications regarding applicability to other cohorts, or may instead represent a dietary trend in IBD patients different to the general population. Moreover, current evidence suggests that certain patients may benefit from a low-emulsifier diet as shown by the ADDapt trial in mild-moderate cases of CD, which aligns with the reduction of UPF consumption in IBD.^32^

Importantly, all significant dietary associations in our cohort were observed in patients with UC but not in CD. This finding likely relates to the pathophysiological differences between the two conditions.^33^ Additionally, substantially greater genetic and phenotypic heterogeneity is seen in CD,^3^ which may account for some of the lack of associations seen. For example, we were underpowered to look at ileal versus colonic disease in relation to UPF and dietary fibre intake. As a sensitivity analysis, we considered only subjects with an FC <250 µg/g at diagnosis, as a proxy for disease severity. However, the key findings did not substantially differ (online supplemental table S6).

One of the key clinical findings was that baseline FC was strongly associated with both patient-reported and objective flares in CD and UC. These data are in keeping with the known disconnect between mucosal inflammation and symptoms seen in IBD,^34^ and that even in perceived clinical remission, mucosal inflammation may persist, increasing risk of flare. These findings reinforce the importance of incorporating objective and sensitive biomarkers like FC into monitoring strategies. The CALM study demonstrated that a treat-to-target strategy,^35^ using a FC target of <250 µg/g, resulted in improved mucosal healing outcomes in patients with CD. Following this pivotal trial, FC values of <250 µg/g are now widely adopted as treatment targets in real-world practice. Although our study was not interventional in nature, when looking at FC cut-offs, our data suggest that even more stringent targets may be required, as patients with FC <50 µg/g had a significantly lower risk of flare when compared with both FC 50–250 µg/g and FC >250 µg/g (figure 3).

We identified differences in associations with patient-reported flares versus objective flares. Female sex, lower haemoglobin levels and lower IBD-Control 8 and VAS scores were associated with an increased risk of patient-reported flares but not objective flares. These findings suggest that subjective disease burden may be influenced by non-inflammatory factors, including functional symptoms such as those seen in irritable bowel syndrome, which is more prevalent in women.^36^ Additionally, anaemia-related fatigue may contribute to symptom perception, independent of mucosal inflammation.^37^ The distinction between patient-reported flares and objective flares is a notable strength of this study. Our findings suggest that some self-reported flares may represent early or subclinical inflammation, as indicated by elevated FC levels, while others may reflect functional symptoms not driven by inflammation.

The PREdiCCt study has several strengths. It is the largest and most comprehensive prospective study of its kind, with 2629 IBD patients recruited from 47 centres, of whom 1091 had both baseline FFQ and FC data, and a long-term follow-up of >4 years. We used validated dietary assessment tools and objective biomarkers to evaluate flare risk. Importantly, all patients were in self-reported clinical remission at baseline, allowing us to explore associations with flare in a stable cohort. Indeed, this makes our data broadly generalisable to the majority of IBD patients attending routine clinic follow-up in the real world.

Limitations of this study include the lack of any protocolised mucosal assessment at baseline or during follow-up. Completion of follow-up questionnaires via our e-portal was variable and partly explained by the limitations of the legacy software available to us at study launch. Study set-up and database construction was done between 2015 and 2016, before the era of routine remote assessment of patient-reported outcomes. Furthermore, we performed a robust end of study phenotyping exercise to ensure all relevant data points were collected during follow-up. Additionally, not all patients completed the FFQ at baseline, although it should be noted that there were only minimal differences between the characteristics of those with and without dietary assessment. The study was partially conducted during the COVID-19 pandemic. In addition to halting recruitment in March 2020, pandemic-related changes, such as treatment discontinuation due to fear of infection^38^ or changes in diet and lifestyle, may have influenced our findings. These factors should be considered when interpreting the results and comparing them to prepandemic cohorts. Due to the amount of data collected, many statistical tests were performed, increasing the risk of type I errors (online supplemental table S7 presents Bonferroni adjusted p values for variables which were not prespecified as primary exposures).

In conclusion, our study provides robust prospective evidence that habitual diet, particularly meat consumption, may influence flare risk in UC, and that FC is strongly associated with disease flare, even in perceived remission. These findings support the integration of dietary assessments and biomarker-based monitoring into routine IBD care and highlight the need for further research into personalised, preventive strategies.

## Supplementary material

10.1136/gutjnl-2025-337846 online supplemental file 1

10.1136/gutjnl-2025-337846 online supplemental file 2

10.1136/gutjnl-2025-337846 online supplemental file 3

## Data Availability

Data are available upon reasonable request.
