# Supplementary material for: Associations between demographic, clinical and dietary factors and flares in inflammatory bowel disease: the PRognostic effect of Environmental factors in Crohn’s and Colitis (PREdiCCt) prospective cohort study
Source: Gut. 2026 Jan 19;75(8):e337846. doi: 10.1136/gutjnl-2025-337846 (PMC13479245; doi:10.1136/gutjnl-2025-337846)
Supplement: Supplementary file 1 [file gutjnl-75-8-s002.pdf]

Supplementary note for Constantine-Cooke, Gros, and Plevris et al.

Nathan Constantine-Cooke[1, 2]

[1]Centre for Genomic and Experimental Medicine, Institute of Genetics and Cancer, University of Edinburgh, Edinburgh, UK
[2]MRC Human Genetics Unit, Institute of Genetics and Cancer, University of Edinburgh, Edinburgh, UK

Supplemental Note 1: Formal Definition of NOVA intake score

When completing the FFQ, participants were asked to report (a) portion size normally consumed, (b) number of times this portion is consumed in one day and (c) number of days per week food type is consumed. Participant's daily average consumption (in grams) of a food and drink type (x) was calculated by:

$$x = \frac{c}{7}(a + b)$$

Standardised number of portions consumed daily for food and drink type (y) was calculated by dividing consumption (x) by the Foods Standard Agency average UK-portion size (z).

$$y = \frac{x}{z}$$

Nova intake scores (N) were calculated by multiplying the number of standardised portions consumed (y) by their corresponding Nova score (M) assigned in the database. This process is repeated for all 169 food and drink types and totalled to give one overall Nova intake score. This score is a marker representative of UPF intake.

$$N = \sum_{i=1}^{169}(y_i M_i)$$
