# Supplementary material for: Associations between demographic, clinical and dietary factors and flares in inflammatory bowel disease: the PRognostic effect of Environmental factors in Crohn’s and Colitis (PREdiCCt) prospective cohort study
Source: Gut. 2026 Jan 19;75(8):e337846. doi: 10.1136/gutjnl-2025-337846 (PMC13479245; doi:10.1136/gutjnl-2025-337846)
Supplement: Supplementary file 2 [file gutjnl-75-8-s001.docx]

**Supplementary Material**

Analyses across all variables, both baseline and time-to-flare, are available online at [www.constantine-cooke.com/predicct-analysis/](http://www.constantine-cooke.com/predicct-analysis/)

**Supplementary Figures**

*Table of contents*

**Figure S1. Overall study design of the PREdiCCt study**

**Figure S2. Number of responses for each month of follow-up**

**Figure S3 Hazard ratios for Cox models with continuous faecal calprotectin**

**Figure S4. Flares stratified by smoking**

**Figure S5: Forest plot of Cox model for total meat intake, adjusted for smoking status**

**Figure S5.1. Crohn’s disease patient-reported flare**

**Figure S5.2 Crohn’s disease objective flare**

**Figure S5.3 Ulcerative colitis patient-reported flare**

**Figure S5.4 Ulcerative colitis objective flare**

**Figure S6: Forest plot of Cox model for dietary fibre, adjusted for smoking status**

[**Figure S6.1. Crohn’s disease patient-reported flare**](https://docs.google.com/document/u/0/d/1rwIVcybFGOlbQ8yVHMMoGGbRIBiZWKoPeBn4mIMksmA/edit)

**Figure S6.2 Crohn’s disease objective flare**

**Figure S6.3 Ulcerative colitis patient-reported flare**

**Figure S6.4 Ulcerative colitis objective flare**

**Figure S7: Forest plot of Cox model for polyunsaturated fatty acid intake, adjusted for smoking status**

**Figure S7.1. Crohn’s disease patient-reported flare**

**Figure S7.2 Crohn’s disease objective flare**

**Figure S7.3 Ulcerative colitis patient-reported flare**

**Figure S7.4 Ulcerative colitis objective flare**

**Figure S8: Forest plot of Cox model for ultra processed food intake, adjusted for smoking status**

**Figure S8.1. Crohn’s disease patient-reported flare**

**Figure S8.2 Crohn’s disease objective flare**

**Figure S8.3 Ulcerative colitis patient-reported flare**

**Figure S8.4 Ulcerative colitis objective flare**

**Figure S9. Disease duration of PREdiCCt cohort stratified by IBD type**

**Figure S10. Distribution of month of recruitment of PREdiCCt participants**

**Figure S11. Dietary distribution of the FFQ subcohort expressed as percentages of energy intake**

**Figure S12. Diet quality index for the FFQ cohort, stratified by IBD type**

**Figure S13. Flares stratified by sex**

**Figure S14. Flares stratified by index of multiple deprivation**

**Figure S15. Objective flares in ulcerative colitis stratified by meat categories**

**Figure S16. Hazard ratio (HR) for total meat intake obtained from a Cox model fitted to objective flare in ulcerative colitis/IBDU**

**Figure S17. Flares stratified by quartiles of dietary fibre**

**Figure S18. Flares stratified by polyunsaturated fatty acid (PUFA) intake**

**Figure S19. Percentage of calorie intake attributable to ultra-processed food**

**Figure S20. Distribution of C-reactive protein by faecal calprotectin subgroups**

**Figure S1.** Overall study design of the PREdiCCt study

**Figure S2.** Number of responses for each month of follow-up for (A) the whole PREdiCCt cohort, (B) the faecal calprotectin cohort, and (C) the food frequency questionnaire cohort.

**Figure S3.** Hazard ratios (HR) for faecal calprotectin (FC) obtained using Cox models with (log) FC modelled continuously using natural cubic splines. (A) Crohn’s disease patient-reported flare, (B) Crohn’s disease objective flare, (C) Ulcerative colitis/IBDU patient-reported flare, and (D) Ulcerative colitis/IBDU objective flare. Additional analyses which consider faecal calprotectin as a continuous variable are available online: <https://www.constantine-cooke.com/predicct-analysis/Survival/FC%20continuous/Diet.html>

**Figure S4.** Log-rank tests of Kaplan-Meier curves stratified by smoking status for (A) patient-reported flares in Crohn’s disease; (B) objective flares in Crohn’s disease; (C) patient-reported flares in ulcerative colitis; and (D) objective flares in ulcerative colitis.

**Figure S5.1.** Forest plot of Cox model for total meat intake, adjusted for smoking status (imputed via MICE), for patient-reported flares in Crohn’s disease.

# Figure S5.2. Forest plot of Cox model for total meat intake, adjusted for smoking status (imputed via MICE), for objective flares in Crohn’s disease.

**Figure S5.3.** Forest plot of Cox model for total meat intake, adjusted for smoking status (imputed via MICE), for patient-reported flares in ulcerative colitis/inflammatory bowel disease unclassified.

**Figure S5.4.** Forest plot of Cox model for total meat intake, adjusted for smoking status (imputed via MICE), for objective flares in ulcerative colitis/inflammatory bowel disease unclassified.

**Figure S6.1.** Forest plot of Cox model for dietary fibre, adjusted for smoking status (imputed via MICE), for patient-reported flares in Crohn’s disease.

# Figure S6.2. Forest plot of Cox model for dietary fibre, adjusted for smoking status (imputed via MICE), for objective flares in Crohn’s disease.

**Figure S6.3.** Forest plot of Cox model for dietary fibre, adjusted for smoking status (imputed via MICE), for patient-reported flares in ulcerative colitis/inflammatory bowel disease unclassified.

**Figure S6.4.** Forest plot of Cox model for dietary fibre, adjusted for smoking status (imputed via MICE), for objective flares in ulcerative colitis/inflammatory bowel disease unclassified.

**Figure S7.1.** Forest plot of Cox model for polyunsaturated fatty acid intake, adjusted for smoking status (imputed via MICE), for patient-reported flares in Crohn’s disease.

# Figure S7.2. Forest plot of Cox model for polyunsaturated fatty acid intake, adjusted for smoking status (imputed via MICE), for objective flares in Crohn’s disease.

**Figure S7.3.** Forest plot of Cox model for polyunsaturated fatty acid intake, adjusted for smoking status (imputed via MICE), for patient-reported flares in ulcerative colitis/inflammatory bowel disease unclassified.

**Figure S7.4.** Forest plot of Cox model for polyunsaturated fatty acid intake, adjusted for smoking status (imputed via MICE), for objective flares in ulcerative colitis/inflammatory bowel disease unclassified.

**Figure S8.1.** Forest plot of Cox model for ultra processed food (NOVA Score 4) intake, adjusted for smoking status (imputed via MICE), for patient-reported flares in Crohn’s disease.

# Figure S8.2. Forest plot of Cox model for ultra processed food (NOVA Score 4) intake, adjusted for smoking status (imputed via MICE), for objective flares in Crohn’s disease.

**Figure S8.3.** Forest plot of Cox model for ultra processed food (NOVA Score 4) intake, adjusted for smoking status (imputed via MICE), for patient-reported flares in ulcerative colitis/inflammatory bowel disease unclassified.

**Figure S8.4.** Forest plot of Cox model for ultra processed food (NOVA Score 4) intake, adjusted for smoking status (imputed via MICE), for objective flares in ulcerative colitis/inflammatory bowel disease unclassified.

**Figure S9.** Disease duration of PREdiCCt cohort stratified by IBD type

**Figure S10.** Distribution of month of recruitment of PREdiCCt participants

**Figure S11.** Dietary distribution of the FFQ subcohort expressed as percentages of energy intake and stratified by sex and diagnosis of either Crohn’s disease (CD) or ulcerative colitis (UC)/inflammatory bowel disease (IBD) unclassified.

**Figure S12.** Diet quality index for the FFQ cohort, stratified by IBD type

# Figure S13. Flares stratified by sex (A) patient-reported flares in Crohn’s disease; (B) objective flares in Crohn’s disease; (C) patient-reported flares in ulcerative colitis; and (D) objective flares in ulcerative colitis.

# Figure S14. Flares stratified by index of multiple deprivation (IMD) (A) patient-reported flares in Crohn’s disease; (B) objective flares in Crohn’s disease; (C) patient-reported flares in ulcerative colitis; and (D) objective flares in ulcerative colitis.

**Figure S15**. Objective flares in ulcerative colitis stratified by (A) meat; (B) unprocessed red meat; and (C) unprocessed white meat

**Figure S16.** Hazard ratio (HR) for total meat intake obtained from a Cox model fitted to objective flare in ulcerative colitis/IBDU. Total meat intake and faecal calprotectin were modelled continuously using natural cubic splines

# Figure S17. Flares stratified by quartiles of dietary fibre. (A) patient-reported flares in Crohn’s disease; (B) objective flares in Crohn’s disease; (C) patient-reported flares in ulcerative colitis; and (D) objective flares in ulcerative colitis.

# Figure S18. Flares stratified by quartiles of polyunsaturated fatty acid (PUFA) intake. (A) patient-reported flares in Crohn’s disease; (B) objective flares in Crohn’s disease; (C) patient-reported flares in ulcerative colitis; and (D) objective flares in ulcerative colitis.

**Figure S19.** Percentage of calorie intake attributable to ultra-processed food (NOVA score of 4) (A) patient-reported flares in Crohn’s disease; (B) objective flares in Crohn’s disease; (C) patient-reported flares in ulcerative colitis; and (D) objective flares in ulcerative colitis.

# Figure S20. Distribution of C-reactive protein by faecal calprotectin subgroups.

**Supplementary Tables**

*Table of contents*

**Table S1. Recruitment sites and associated investigators/research nurses**

**Table S2. Baseline phenotyping and laboratory tests processed locally as part of routine annual work-up**

**Table S3. Environmental and lifestyle questionnaires utilised in PREdiCCt**

**Table S4. Recorded IBD treatments subdivided by subcohorts**

**Table S5. Comparison by FC subgroups subdivided by IBD types**

**Table S6. Parameter estimates for dietary risk factors in main and sensitivity (CALPRO < 250) analyses**

**Table S7. P-values for dietary risk factors, and adjusted p-values for exploratory risk factors**

**Table S1.** Recruitment sites and associated investigators/research nurses.

| **Site** | **Title** | **Investigator** | **Nurse/s** |
| --- | --- | --- | --- |
| Western General Hospital, Edinburgh | Prof | Charlie Lees | Judy Coyle  Debbie Alexander  Kate Covil |
| Glasgow Royal Infirmary | Prof | Daniel Gaya | Lynne Turner |
| Royal Hospital for Children and Young People, Edinburgh | Dr | Paul Henderson | Margaret Millar |
| Queen Elizabeth University Hospital, Glasgow | Dr | Jonathan Macdonald | Faye McMeeken  Shona Finan |
| Aberdeen Royal Infirmary | Dr | Malcolm Smith | Siby Joseph |
| Ninewells Hospital, Dundee | Dr | Craig Mowat | Samer Mohammed  Anne Alliott |
| Raigmore Hospital, Inverness | Dr | Angus Watson | Rachael Campbell |
| Royal Hospital for Children, Glasgow | Dr | Richard Hansen | Lisa Gervais |
| Royal Infirmary of Edinburgh | Prof | Charlie Lees | Garry Tucker |
| St John’s Hospital, Livingston | Prof | Charlie Lees |  |
| Lanarkshire | Dr | Muhhammad Akhtar | Karen Leitch |
| South Eastern Health Belfast | Dr | Patrick Allen | Victoria Adell |
| Royal Free Hospital | Dr | Charles Murray | Glykeria Pakou  Eric Witele |
| Cambridge University Hospitals | Dr | Miles Parkes | Konstantina Strongili |
| Royal Devon and Exeter | Dr | Nicholas Kennedy | Hannah Mabb  Melanie Hutchings |
| Royal London Hospital | Dr | James Lindsay | Irish Lee  Bessie Cipriano |
| Fife | Dr | Sian Gilchrist | Fleur Davey |
| Sandwell West Birmingham | Dr | Nigel Trudgill | Samia Hussain  Rita Kumar |
| Forth Valley | Dr | Stuart Paterson | Anne Todd |
| Queen Elizabeth Kings Lynn | Ms | Fran Bredin | Hannah Bloxham |
| Buckinghamshire (Stoke Mandeville) | Ms | Ruth Penn | Ruth Penn |
| Taunton and Somerset | Ms | Alison Moss | Jacob Lucas |
| Pinderfield Hospital | Dr | Deven Vani | Julie Burton  Patricia Kane  Stephen Lupton  Elizabeth Denis |
| University Hospital Coventry | Prof | Chuka Nwokolo | Sandra Stringer |
| Kingston Hospital | Ms | Fiona Donovan | Rosie Herdmangrant |
| Gateshead | Dr | Jamie Barbour | Maureen Armstrong  Ann Wilson |
| Royal Hampshire Country Hospital | Dr | John Gordon | Emma Levell |
| King's College | Dr | Alexandra Kent | Lee Meng Choong |
| Bronglais General Hospital | Dr | Mark Narain | Ronda Loosley  Sarah Jones  Rebecca Woolf Robert  Heather McGuinness |
| Bristol Royal Infirmary | Dr | Tom Creed | Moira Tait |
| Withybush General Hospital | Ms | Kerrie Johns | Catherine Macphee  Hachel Hughes  Helen Thomas  Abigail Taylor |
| Salford Royal Hospital | Prof | John McLaughlin | Anne Keen |
| West Suffolk Hospital | Dr | Dan Sharpstone | Angharad Williams  Joanne Kellet  Abbie Holden |
| Eastbourne General Hospital | Ms | Judith Tidbury | Claire Rutherfurd |
| County Durham and Darlington | Dr | Anjan Dhar | Ellen Brown |
| Guy's and St Thomas | Dr | Peter Irving | Jessica Cordle  Zareen Bheekhun |
| Nottingham Queen's Medical Centre | Ms | Emma Connor | Emma Chamberlain |
| Warrington and Halton Hospital | Dr | Timi Patani | Lisa Ditchfield |
| Royal Berkshire Hospital | Ms | Susanna Malkakorpi | Sarosh Kymani |
| Kettering General Hospital | Dr | Ajay Verma | Parizade Raymode |
| Liverpool Royal | Dr | Alan Steel | Martina Lofthouse |
| Glangwili General Hospital | Dr | Aashish Kumar | Rachel Hughes |
| Newcastle Royal Victoria Infirmary | Dr | Chris Lamb | Lesley Jeffrey  Mary Doona |
| University Hospital of Wales, Cardiff | Dr | Dharmaraj Durai | Liz Harmer Griebel |
| London North West | Prof | Ailsa Hart | Sambasivarao Gurram |
| Royal Surrey | Dr | Christopher Alexakis | Joanna Smith  Asharee Green |
| James Paget | Dr | Paul Banim | Helen Sutherland |
| St Georges | Dr | Kamal Patel | Abigail Seward |
| Maidstone | Ms | Eleanor Warner | Banher Sandhu  Maureen Williams |
| Aintree | Dr | Suhail Ahmed | Lilian Wajero |

**Table S2.** Baseline phenotyping and laboratory tests processed locally as part of routine annual work-up

| **Demographic and Phenotypic Data** | Date of birth, sex, age, BMI, Montreal classification (location / behaviour), date of diagnosis, current / prior drug therapy (IBD and non-IBD), surgical interventions, family history and smoking status |
| --- | --- |
| **Routine Biochemistry** | FBC, urea & electrolytes, LFTs, albumin, C-reactive protein, ferritin, B12, folate and Vit D |

**Table S3.** Environmental and lifestyle questionnaires utilised in PREdiCCt

**List of Questionnaires**

IBD Questionnaire (IBDQ)

The Crohn’s and Ulcerative Colitis Questionnaire - 8 (CUCQ-8)

Pittsburgh Sleep Quality Index (PSQI)

Hospital Anxiety and Depression Scale (HADS)

Patient Health Questionnaire-15 (PHQ-15)

Short Form Health Survey Questionnaire (SF-12)

Holmes and Rahe Stress Scale (Top 20)

Malnutrition Universal Screening Tool (MUST)

COVID-19 Questionnaire

Global Physical Activity Questionnaire (GPAQ-2)

Fast Alcohol Screening Tool (FAST)

Fatigue, urgency, leakage and bowel control (IBD-Boost Survey)

Connor-Davidson Resilience Scale 25 (CD-RISC-25)

**Table S4.1** Recorded IBD treatments across the full cohort ongoing at recruitment. N denotes the number of subjects recorded as having received at least one medication, either at recruitment or previously prescribed. Due to patients either receiving concomitant therapies or having stopped all treatments, the number of prescribed medications do not sum up to N.

|  | **Crohn’s disease (N=1126)** | **Ulcerative colitis/IBDU**  **(N=991)** |
| --- | --- | --- |
| **5-ASA** | 89 (7.9%) | 551 (55.6%) |
| **Azathioprine** | 325 (28.9%) | 218 (22.0%) |
| **Mercaptopurine** | 93 (8.3%) | 64 (6.5%) |
| **Methotrexate** | 61 (5.4%) | 14 (1.4%) |
| **Ciclosporin** | 1 (0.1%) | 1 (0.1%) |
| **Infliximab** | 286 (25.4%) | 104 (10.5%) |
| **Vedolizumab** | 65 (5.8%) | 102 (10.3%) |
| **Adalimumab** | 164 (14.6%) | 29 (2.9%) |
| **Ustekinumab** | 14 (1.2%) | 3 (0.3%) |
| **Golimumab** | 0 (0.0%) | 6 (0.6%) |

**Table S4.2** Recorded ongoing IBD treatments across the faecal calprotectin cohort ongoing at recruitment. N denotes the number of subjects recorded as having received at least one medication, either at recruitment or previously prescribed. Due to patients either receiving concomitant therapies or having stopped all treatments, the number of prescribed medications do not sum up to N.

|  | **Crohn’s disease (N=931)** | **Ulcerative colitis/IBDU**  **(N=801)** |
| --- | --- | --- |
| **5-ASA** | 75 (8.1%) | 436 (54.4%) |
| **Azathioprine** | 270 (29.0%) | 177 (22.1%) |
| **Mercaptopurine** | 78 (8.4%) | 51 (6.4%) |
| **Methotrexate** | 52 (5.6%) | 12 (1.5%) |
| **Ciclosporin** | 1 (0.1%) | 1 (0.1%) |
| **Infliximab** | 241 (25.9%) | 87 (10.9%) |
| **Vedolizumab** | 55 (5.9%) | 90 (11.2%) |
| **Adalimumab** | 137 (14.7%) | 21 (2.6%) |
| **Ustekinumab** | 10 (1.1%) | 3 (0.4%) |
| **Golimumab** | 0 (0.0%) | 5 (0.6%) |

**Table S4.3** Recorded ongoing IBD treatments across the food frequency questionnaire cohort ongoing at recruitment. N denotes the number of subjects recorded as having received at least one medication, either at recruitment or previously prescribed. Due to patients either receiving concomitant therapies or having stopped all treatments, the number of prescribed medications do not sum up to N.

|  | **Crohn’s disease (N=403)** | **Ulcerative colitis/IBDU**  **(N=382)** |
| --- | --- | --- |
| **5-ASA** | 30 (7.4%) | 223 (58.4%) |
| **Azathioprine** | 111 (27.5%) | 79 (20.7%) |
| **Mercaptopurine** | 32 (7.9%) | 23 (6.0%) |
| **Methotrexate** | 16 (4.0%) | 4 (1.0%) |
| **Ciclosporin** | 0 (0.0%) | 0 (0.0%) |
| **Infliximab** | 81 (20.1%) | 27 (7.1%) |
| **Vedolizumab** | 19 (4.7%) | 29 (7.6%) |
| **Adalimumab** | 69 (17.1%) | 13 (3.4%) |
| **Ustekinumab** | 3 (0.7%) | 2 (0.5%) |
| **Golimumab** | 0 (0.0%) | 4 (1.0%) |

**Table S5.1** FC cohort sub-divided by IBD type: Crohn’s disease

|  | **FC < 50**  **(N=533)** | **FC 50-250**  **(N=381)** | **FC > 250**  **(N=204)** | **p-value** |
| --- | --- | --- | --- | --- |
| **Disease Duration (years)** |  |  |  | 0.54 |
| Median (IQR) | 10.2 (4.9 - 20.9) | 10.6 (5.0 - 20.2) | 10.1 (5.4 - 17.9) |  |
| Missing | 33 (6.2%) | 21 (5.5%) | 10 (4.9%) |  |
| **Montreal Location** |  |  |  | 0.61 |
| L1 | 138 (25.9%) | 99 (26.0%) | 46 (22.5%) |  |
| L2 | 128 (24.0%) | 90 (23.6%) | 60 (29.4%) |  |
| L3 | 176 (33.0%) | 136 (35.7%) | 73 (35.8%) |  |
| L4 only | < 5 | < 5 | < 5 |  |
| Missing | 88 (16.5%) | 52 (13.6%) | 22 (10.8%) |  |
| **Montreal L4 Presence** |  |  |  | 0.06 |
| Not present | 398 (74.7%) | 293 (76.9%) | 151 (74.0%) |  |
| Present | 47 (8.8%) | 36 (9.4%) | 31 (15.2%) |  |
| Missing | 88 (16.5%) | 52 (13.6%) | 22 (10.8%) |  |
| **Montreal Behaviour** |  |  |  | 0.27 |
| B1 | 279 (52.3%) | 211 (55.4%) | 127 (62.3%) |  |
| B2 | 96 (18.0%) | 73 (19.2%) | 37 (18.1%) |  |
| B3 | 51 (9.6%) | 31 (8.1%) | 11 (5.4%) |  |
| Missing | 107 (20.1%) | 66 (17.3%) | 29 (14.2%) |  |
| **Perianal** |  |  |  | 0.52 |
| Yes | 149 (28.0%) | 106 (27.8%) | 66 (32.4%) |  |
| No | 283 (53.1%) | 224 (58.8%) | 112 (54.9%) |  |
| Missing | 101 (18.9%) | 51 (13.4%) | 26 (12.7%) |  |
| **Harvey-Bradshaw Index** |  |  |  | 0.31 |
| Median (IQR) | 2.00 (1.00 - 4.00) | 2.00 (1.00 - 4.00) | 2.00 (1.00 - 3.00) |  |
| Missing | 331 (62.1%) | 227 (59.6%) | 116 (56.9%) |  |
| **PRO2** |  |  |  |  |
| Median (IQR) | 0 (0 - 2.00) | 0 (0 - 2.00) | 0 (0 - 1.00) |  |
| Missing | 242 (45.4%) | 135 (35.4%) | 69 (33.8%) |  |
| **Surgery** |  |  |  | **0.025** |
| Yes | 251 (47.1%) | 193 (50.7%) | 78 (38.2%) |  |
| No | 262 (49.2%) | 181 (47.5%) | 118 (57.8%) |  |
| Missing | 20 (3.8%) | 7 (1.8%) | 8 (3.9%) |  |
| **Smoking** |  |  |  | **0.026** |
| Current | 32 (6.0%) | 26 (6.8%) | 5 (2.5%) |  |
| Previous | 153 (28.7%) | 108 (28.3%) | 41 (20.1%) |  |
| Never | 234 (43.9%) | 169 (44.4%) | 105 (51.5%) |  |
| Missing | 114 (21.4%) | 78 (20.5%) | 53 (26.0%) |  |
| **ECigarettes** |  |  |  | 0.20 |
| Current | 19 (3.6%) | 18 (4.7%) | < 5 |  |
| Previous | 16 (3.0%) | 13 (3.4%) | < 5 |  |
| Never | 382 (71.7%) | 272 (71.4%) | 145 (71.1%) |  |
| Missing | 116 (21.8%) | 78 (20.5%) | 53 (26.0%) |  |

**Table S5.2** FC cohort sub-divided by IBD type: Ulcerative Colitis / IBDU

|  | **FC < 50**  **(N=544)** | **FC 50-250**  **(N=302)** | **FC > 250**  **(N=180)** | **p-value** |
| --- | --- | --- | --- | --- |
| **Disease Duration (years)** |  |  |  | **0.026** |
| Median (IQR) | 10.1  (4.5 - 17.8) | 8.3 (4.3 - 15.4) | 7.5 (3.1 - 13.4) |  |
| Missing | 27 (5.0%) | 17 (5.6%) | 12 (6.7%) |  |
| **Montreal Extent** |  |  |  | 0.68 |
| E1 | 74 (13.6%) | 34 (11.3%) | 20 (11.1%) |  |
| E2 | 214 (39.3%) | 126 (41.7%) | 76 (42.2%) |  |
| E3 | 126 (23.2%) | 79 (26.2%) | 48 (26.7%) |  |
| Missing | 130 (23.9%) | 63 (20.9%) | 36 (20.0%) |  |
| **Partial Mayo Score** |  |  |  | **<0.001** |
| Median (IQR) | 0 (0 - 1.00) | 0 (0 - 1.00) | 0.500 (0 - 2.00) |  |
| Missing | 171 (31.4%) | 62 (20.5%) | 58 (32.2%) |  |
| **PRO2** |  |  |  |  |
| Median (IQR) | 0 (0 - 1.00) | 0 (0 - 1.00) | 0 (0 - 1.00) |  |
| Missing | 172 (31.6%) | 64 (21.2%) | 59 (32.8%) |  |
| **Smoking** |  |  |  | **0.044** |
| Current | 32 (5.9%) | 10 (3.3%) | 4 (2.2%) |  |
| Previous | 156 (28.7%) | 105 (34.8%) | 64 (35.6%) |  |
| Never | 253 (46.5%) | 131 (43.4%) | 73 (40.6%) |  |
| Missing | 103 (18.9%) | 56 (18.5%) | 39 (21.7%) |  |
| **ECigarettes** |  |  |  | **0.020** |
| Current | 9 (1.7%) | 15 (5.0%) | 7 (3.9%) |  |
| Previous | 15 (2.8%) | 3 (1.0%) | 2 (1.1%) |  |
| Never | 417 (76.7%) | 228 (75.5%) | 131 (72.8%) |  |
| Missing | 103 (18.9%) | 56 (18.5%) | 40 (22.2%) |  |

**Table S6.1** Parameter estimates for risk factors for patient-reported flare in Crohn’s disease, as inferred in univariate analyses (adjusting for known risk factors), in main and sensitivity analyses. CALPRO < 250

|  | **Main (SE)** | **Sensitivity (SE)** |
| --- | --- | --- |
| Meat protein 2nd quartile | -0.003 (0.212) | 0.069 (0.246) |
| Meat protein 3rd quartile | -0.143 (0.215) | -0.029 (0.245) |
| Meat protein 4th quartile | -0.075 (0.226) | -0.085 (0.266) |
| Overall meat intake 2nd quartile | -0.028 (0.214) | 0.181 (0.244) |
| Overall meat intake 3rd quartile | -0.114 (0.223) | -0.035 (0.258) |
| Overall meat intake 4th quartile | -0.114 (0.242) | -0.033 (0.277) |
| Overall fish intake 2nd quartile | -0.525 (0.229) | -0.324 (0.262) |
| Overall fish intake 3rd quartile | -0.379 (0.218) | -0.177 (0.248) |
| Overall fish intake 4th quartile | -0.144 (0.215) | -0.078 (0.251) |
| Dietary fibre 2nd quartile | -0.286 (0.220) | -0.245 (0.257) |
| Dietary fibre 3rd quartile | -0.109 (0.222) | -0.178 (0.259) |
| Dietary fibre 4th quartile | -0.043 (0.240) | -0.152 (0.272) |
| PUFA 2nd quartile | -0.284 (0.214) | -0.250 (0.245) |
| PUFA 3rd quartile | -0.426 (0.220) | -0.611 (0.261) |
| PUFA 4th quartile | -0.067 (0.199) | -0.051 (0.227) |
| NOVA Score 2 | -0.060 (0.220) | -0.091 (0.266) |
| NOVA Score 3 | 0.113 (0.215) | 0.189 (0.252) |
| NOVA Score 4 | 0.041 (0.216) | 0.118 (0.253) |
| %UPF 2nd quartile | 0.113 (0.226) | 0.101 (0.274) |
| %UPF 3rd quartile | 0.147 (0.223) | 0.331 (0.263) |
| %UPF 4th quartile | -0.026 (0.231) | 0.167 (0.273) |
| Bread intake 2nd quartile | -0.380 (0.218) | -0.445 (0.254) |
| Bread intake 3rd quartile | -0.276 (0.219) | -0.399 (0.252) |
| Bread intake 4th quartile | -0.185 (0.215) | -0.164 (0.244) |
| Sweet intake 2nd quartile | 0.059 (0.211) | 0.194 (0.248) |
| Sweet intake 3rd quartile | -0.215 (0.227) | -0.107 (0.265) |
| Sweet intake 4th quartile | 0.036 (0.227) | 0.339 (0.263) |
| Drink intake 2nd quartile | -0.006 (0.223) | -0.160 (0.265) |
| Drink intake 3rd quartile | -0.096 (0.222) | 0.040 (0.251) |
| Drink intake 4th quartile | -0.261 (0.210) | -0.313 (0.249) |
| Processed meat intake 2nd quartile | 0.030 (0.231) | -0.209 (0.273) |
| Processed meat intake 3rd quartile | 0.235 (0.228) | 0.287 (0.253) |
| Processed meat intake 4th quartile | 0.152 (0.251) | 0.088 (0.283) |
| Processed plant intake above median | 0.147 (0.177) | 0.167 (0.197) |
| Fruit intake 2nd quartile | 0.118 (0.224) | 0.061 (0.257) |
| Fruit intake 3rd quartile | 0.054 (0.229) | 0.115 (0.261) |
| Fruit intake 4th quartile | 0.049 (0.259) | -0.297 (0.298) |
| Vegetable intake 2nd quartile | -0.017 (0.222) | -0.114 (0.262) |
| Vegetable intake 3rd quartile | -0.225 (0.234) | -0.333 (0.267) |
| Vegetable intake 4th quartile | -0.007 (0.240) | -0.087 (0.274) |
| Red meat intake 2nd tertile | -0.172 (0.192) | -0.238 (0.219) |
| Red meat intake 3rd tertile | -0.090 (0.194) | -0.105 (0.227) |
| White meat intake 2nd quartile | -0.180 (0.226) | -0.091 (0.260) |
| White meat intake 3rd quartile | -0.268 (0.237) | -0.128 (0.265) |
| White meat intake 4th quartile | -0.070 (0.218) | -0.037 (0.255) |
| White fish intake 2nd quartile | -0.747 (0.235) | -0.578 (0.271) |
| White fish intake 3rd quartile | -0.434 (0.223) | -0.230 (0.255) |
| White fish intake 4th quartile | -0.229 (0.216) | -0.175 (0.255) |

**Table S6.2** Parameter estimates for risk factors for objective flare in Crohn’s disease, as inferred in univariate analyses (adjusting for known risk factors), in main and sensitivity analyses.

|  | **Main (SE)** | **Sensitivity (SE)** |
| --- | --- | --- |
| Meat protein 2nd quartile | 0.265 (0.284) | 0.033 (0.348) |
| Meat protein 3rd quartile | 0.175 (0.283) | 0.250 (0.321) |
| Meat protein 4th quartile | -0.032 (0.299) | -0.173 (0.355) |
| Overall meat intake 2nd quartile | -0.135 (0.282) | -0.041 (0.325) |
| Overall meat intake 3rd quartile | -0.098 (0.288) | -0.369 (0.348) |
| Overall meat intake 4th quartile | -0.162 (0.308) | -0.221 (0.354) |
| Overall fish intake 2nd quartile | -0.817 (0.308) | -0.712 (0.372) |
| Overall fish intake 3rd quartile | -0.528 (0.290) | -0.093 (0.326) |
| Overall fish intake 4th quartile | -0.265 (0.273) | -0.182 (0.331) |
| Dietary fibre 2nd quartile | 0.025 (0.291) | -0.151 (0.349) |
| Dietary fibre 3rd quartile | 0.368 (0.291) | 0.138 (0.343) |
| Dietary fibre 4th quartile | 0.329 (0.313) | 0.106 (0.359) |
| PUFA 2nd quartile | 0.100 (0.287) | -0.134 (0.360) |
| PUFA 3rd quartile | 0.092 (0.288) | 0.084 (0.336) |
| PUFA 4th quartile | 0.263 (0.280) | 0.415 (0.324) |
| NOVA Score 2 | 0.404 (0.280) | 0.514 (0.347) |
| NOVA Score 3 | 0.318 (0.295) | 0.386 (0.352) |
| NOVA Score 4 | 0.044 (0.295) | -0.041 (0.373) |
| %UPF 2nd quartile | 0.017 (0.279) | -0.081 (0.334) |
| %UPF 3rd quartile | -0.488 (0.298) | -0.591 (0.361) |
| %UPF 4th quartile | -0.152 (0.283) | -0.089 (0.332) |
| Bread intake 2nd quartile | -0.392 (0.276) | -0.234 (0.318) |
| Bread intake 3rd quartile | -0.370 (0.285) | -0.590 (0.348) |
| Bread intake 4th quartile | -0.491 (0.288) | -0.509 (0.342) |
| Sweet intake 2nd quartile | -0.291 (0.281) | -0.665 (0.342) |
| Sweet intake 3rd quartile | -0.259 (0.278) | -0.366 (0.313) |
| Sweet intake 4th quartile | -0.070 (0.285) | -0.258 (0.331) |
| Drink intake 2nd quartile | -0.078 (0.280) | -0.356 (0.341) |
| Drink intake 3rd quartile | -0.307 (0.289) | -0.570 (0.333) |
| Drink intake 4th quartile | -0.351 (0.272) | -0.658 (0.326) |
| Processed meat intake 2nd quartile | 0.039 (0.301) | -0.274 (0.368) |
| Processed meat intake 3rd quartile | 0.216 (0.287) | 0.272 (0.324) |
| Processed meat intake 4th quartile | -0.151 (0.332) | -0.189 (0.386) |
| Processed plant intake above median | 0.138 (0.229) | 0.184 (0.263) |
| Fruit intake 2nd quartile | -0.342 (0.305) | -0.675 (0.383) |
| Fruit intake 3rd quartile | -0.012 (0.285) | -0.002 (0.335) |
| Fruit intake 4th quartile | -0.153 (0.327) | -0.010 (0.372) |
| Vegetable intake 2nd quartile | -0.083 (0.288) | -0.304 (0.357) |
| Vegetable intake 3rd quartile | -0.132 (0.303) | -0.112 (0.354) |
| Vegetable intake 4th quartile | 0.400 (0.311) | 0.484 (0.362) |
| Red meat intake 2nd tertile | -0.174 (0.241) | -0.336 (0.284) |
| Red meat intake 3rd tertile | -0.451 (0.275) | -0.494 (0.321) |
| White meat intake 2nd quartile | -0.619 (0.286) | -0.722 (0.345) |
| White meat intake 3rd quartile | -0.675 (0.296) | -0.633 (0.337) |
| White meat intake 4th quartile | -0.314 (0.265) | -0.478 (0.325) |
| White fish intake 2nd quartile | -1.172 (0.316) | -1.251 (0.405) |
| White fish intake 3rd quartile | -0.651 (0.294) | -0.338 (0.332) |
| White fish intake 4th quartile | -0.279 (0.270) | -0.069 (0.325) |

**Table S6.3** Parameter estimates for risk factors for patient-reported flare in Ulcerative Colitis, as inferred in univariate analyses (adjusting for known risk factors), in main and sensitivity analyses.

|  | **Main (SE)** | **Sensitivity (SE)** |
| --- | --- | --- |
| Meat protein 2nd quartile | 0.160 (0.202) | 0.222 (0.231) |
| Meat protein 3rd quartile | 0.309 (0.203) | 0.251 (0.231) |
| Meat protein 4th quartile | 0.239 (0.210) | 0.225 (0.239) |
| Overall meat intake 2nd quartile | -0.077 (0.202) | -0.190 (0.228) |
| Overall meat intake 3rd quartile | 0.206 (0.202) | 0.108 (0.230) |
| Overall meat intake 4th quartile | 0.072 (0.225) | -0.048 (0.259) |
| Overall fish intake 2nd quartile | 0.170 (0.208) | -0.001 (0.241) |
| Overall fish intake 3rd quartile | 0.136 (0.213) | 0.045 (0.239) |
| Overall fish intake 4th quartile | 0.400 (0.201) | 0.342 (0.231) |
| Dietary fibre 2nd quartile | -0.132 (0.202) | -0.080 (0.233) |
| Dietary fibre 3rd quartile | -0.034 (0.219) | 0.008 (0.255) |
| Dietary fibre 4th quartile | -0.192 (0.222) | 0.180 (0.249) |
| PUFA 2nd quartile | -0.113 (0.200) | -0.204 (0.225) |
| PUFA 3rd quartile | -0.143 (0.199) | -0.090 (0.229) |
| PUFA 4th quartile | 0.164 (0.193) | 0.037 (0.220) |
| NOVA Score 2 | -0.132 (0.194) | -0.024 (0.220) |
| NOVA Score 3 | -0.107 (0.193) | -0.083 (0.225) |
| NOVA Score 4 | -0.149 (0.200) | 0.004 (0.234) |
| %UPF 2nd quartile | -0.513 (0.187) | -0.394 (0.207) |
| %UPF 3rd quartile | -0.576 (0.199) | -0.525 (0.225) |
| %UPF 4th quartile | -0.533 (0.206) | -0.693 (0.246) |
| Bread intake 2nd quartile | 0.328 (0.185) | 0.266 (0.213) |
| Bread intake 3rd quartile | -0.005 (0.205) | -0.121 (0.232) |
| Bread intake 4th quartile | -0.246 (0.209) | -0.285 (0.239) |
| Sweet intake 2nd quartile | -0.242 (0.196) | -0.027 (0.224) |
| Sweet intake 3rd quartile | -0.279 (0.197) | -0.323 (0.232) |
| Sweet intake 4th quartile | -0.301 (0.194) | -0.363 (0.227) |
| Drink intake 2nd quartile | -0.221 (0.187) | -0.182 (0.209) |
| Drink intake 3rd quartile | -0.091 (0.196) | -0.075 (0.224) |
| Drink intake 4th quartile | -0.152 (0.205) | -0.203 (0.236) |
| Processed meat intake 2nd quartile | -0.313 (0.196) | -0.311 (0.217) |
| Processed meat intake 3rd quartile | -0.200 (0.202) | -0.373 (0.237) |
| Processed meat intake 4th quartile | -0.222 (0.215) | -0.394 (0.250) |
| Processed plant intake above median | 0.068 (0.164) | 0.292 (0.182) |
| Fruit intake 2nd quartile | 0.087 (0.215) | 0.064 (0.238) |
| Fruit intake 3rd quartile | 0.137 (0.215) | 0.035 (0.245) |
| Fruit intake 4th quartile | 0.090 (0.234) | -0.132 (0.267) |
| Vegetable intake 2nd quartile | 0.089 (0.211) | 0.129 (0.243) |
| Vegetable intake 3rd quartile | 0.238 (0.213) | 0.331 (0.245) |
| Vegetable intake 4th quartile | 0.214 (0.223) | 0.382 (0.250) |
| Red meat intake 2nd tertile | 0.180 (0.166) | 0.117 (0.194) |
| Red meat intake 3rd tertile | -0.087 (0.181) | -0.054 (0.202) |
| White meat intake 2nd quartile | 0.077 (0.200) | -0.111 (0.232) |
| White meat intake 3rd quartile | 0.048 (0.190) | 0.023 (0.215) |
| White meat intake 4th quartile | 0.025 (0.206) | -0.107 (0.233) |
| White fish intake 2nd quartile | 0.124 (0.216) | -0.057 (0.247) |
| White fish intake 3rd quartile | 0.098 (0.205) | 0.015 (0.230) |
| White fish intake 4th quartile | 0.464 (0.201) | 0.315 (0.227) |

**Table S6.4** Parameter estimates for risk factors for objective flare in Ulcerative Colitis, as inferred in univariate analyses (adjusting for known risk factors), in main and sensitivity analyses.

|  | **Main (SE)** | **Sensitivity (SE)** |
| --- | --- | --- |
| Meat protein 2nd quartile | 0.334 (0.295) | 0.528 (0.353) |
| Meat protein 3rd quartile | 0.439 (0.292) | 0.535 (0.348) |
| Meat protein 4th quartile | 0.782 (0.290) | 0.918 (0.343) |
| Overall meat intake 2nd quartile | -0.263 (0.307) | -0.518 (0.354) |
| Overall meat intake 3rd quartile | 0.423 (0.282) | 0.388 (0.317) |
| Overall meat intake 4th quartile | 0.667 (0.308) | 0.381 (0.353) |
| Overall fish intake 2nd quartile | -0.221 (0.281) | -0.165 (0.341) |
| Overall fish intake 3rd quartile | -0.141 (0.275) | 0.152 (0.316) |
| Overall fish intake 4th quartile | 0.150 (0.262) | 0.172 (0.328) |
| Dietary fibre 2nd quartile | 0.224 (0.289) | 0.136 (0.329) |
| Dietary fibre 3rd quartile | 0.364 (0.305) | 0.275 (0.349) |
| Dietary fibre 4th quartile | 0.157 (0.306) | 0.172 (0.347) |
| PUFA 2nd quartile | -0.091 (0.261) | -0.232 (0.309) |
| PUFA 3rd quartile | -0.273 (0.268) | -0.164 (0.314) |
| PUFA 4th quartile | -0.143 (0.267) | -0.029 (0.305) |
| NOVA Score 2 | 0.435 (0.276) | 0.358 (0.315) |
| NOVA Score 3 | 0.473 (0.278) | 0.265 (0.322) |
| NOVA Score 4 | 0.369 (0.289) | 0.300 (0.334) |
| %UPF 2nd quartile | -0.575 (0.255) | -0.578 (0.294) |
| %UPF 3rd quartile | -0.791 (0.284) | -0.918 (0.340) |
| %UPF 4th quartile | -0.255 (0.255) | -0.189 (0.287) |
| Bread intake 2nd quartile | 0.264 (0.254) | 0.103 (0.301) |
| Bread intake 3rd quartile | 0.118 (0.268) | 0.148 (0.302) |
| Bread intake 4th quartile | -0.395 (0.294) | -0.351 (0.336) |
| Sweet intake 2nd quartile | -0.369 (0.264) | -0.329 (0.297) |
| Sweet intake 3rd quartile | -0.675 (0.286) | -1.014 (0.352) |
| Sweet intake 4th quartile | -0.113 (0.249) | -0.407 (0.295) |
| Drink intake 2nd quartile | -0.274 (0.275) | -0.382 (0.324) |
| Drink intake 3rd quartile | 0.297 (0.268) | 0.643 (0.295) |
| Drink intake 4th quartile | 0.255 (0.280) | 0.009 (0.338) |
| Processed meat intake 2nd quartile | 0.169 (0.268) | 0.289 (0.307) |
| Processed meat intake 3rd quartile | 0.117 (0.287) | 0.082 (0.338) |
| Processed meat intake 4th quartile | 0.267 (0.301) | 0.343 (0.351) |
| Processed plant intake above median | -0.237 (0.235) | -0.284 (0.280) |
| Fruit intake 2nd quartile | -0.040 (0.286) | -0.032 (0.321) |
| Fruit intake 3rd quartile | -0.117 (0.293) | -0.170 (0.339) |
| Fruit intake 4th quartile | 0.113 (0.305) | -0.037 (0.354) |
| Vegetable intake 2nd quartile | 0.232 (0.292) | 0.054 (0.337) |
| Vegetable intake 3rd quartile | 0.599 (0.286) | 0.541 (0.325) |
| Vegetable intake 4th quartile | 0.391 (0.305) | 0.338 (0.338) |
| Red meat intake 2nd tertile | 0.269 (0.238) | 0.651 (0.281) |
| Red meat intake 3rd tertile | 0.591 (0.230) | 0.930 (0.269) |
| White meat intake 2nd quartile | 0.341 (0.284) | 0.214 (0.331) |
| White meat intake 3rd quartile | 0.320 (0.272) | 0.247 (0.319) |
| White meat intake 4th quartile | 0.644 (0.280) | 0.476 (0.325) |
| White fish intake 2nd quartile | -0.082 (0.275) | 0.068 (0.324) |
| White fish intake 3rd quartile | -0.311 (0.280) | -0.256 (0.334) |
| White fish intake 4th quartile | 0.041 (0.260) | 0.231 (0.313) |

**Table S6.5** Parameter estimates for risk factors for patient-reported flare in Inflammatory Bowel disease, as inferred in univariate analyses (adjusting for known risk factors), in main and sensitivity analyses.

|  | **Main (SE)** | **Sensitivity (SE)** |
| --- | --- | --- |
| Meat protein 2nd quartile | 0.081 (0.146) | 0.160 (0.168) |
| Meat protein 3rd quartile | 0.104 (0.146) | 0.130 (0.167) |
| Meat protein 4th quartile | 0.095 (0.153) | 0.093 (0.177) |
| Overall meat intake 2nd quartile | -0.035 (0.145) | 0.015 (0.165) |
| Overall meat intake 3rd quartile | 0.080 (0.148) | 0.080 (0.170) |
| Overall meat intake 4th quartile | -0.004 (0.163) | -0.019 (0.187) |
| Overall fish intake 2nd quartile | -0.148 (0.153) | -0.160 (0.178) |
| Overall fish intake 3rd quartile | -0.067 (0.151) | -0.008 (0.172) |
| Overall fish intake 4th quartile | 0.158 (0.146) | 0.152 (0.170) |
| Dietary fibre 2nd quartile | -0.172 (0.146) | -0.129 (0.171) |
| Dietary fibre 3rd quartile | -0.060 (0.153) | -0.079 (0.180) |
| Dietary fibre 4th quartile | -0.110 (0.159) | 0.027 (0.182) |
| PUFA 2nd quartile | -0.157 (0.146) | -0.195 (0.166) |
| PUFA 3rd quartile | -0.264 (0.147) | -0.343 (0.171) |
| PUFA 4th quartile | 0.048 (0.139) | -0.023 (0.159) |
| NOVA Score 2 | -0.113 (0.144) | -0.081 (0.168) |
| NOVA Score 3 | -0.017 (0.143) | 0.001 (0.166) |
| NOVA Score 4 | -0.084 (0.146) | -0.005 (0.170) |
| %UPF 2nd quartile | -0.192 (0.141) | -0.151 (0.161) |
| %UPF 3rd quartile | -0.217 (0.144) | -0.101 (0.163) |
| %UPF 4th quartile | -0.300 (0.151) | -0.302 (0.175) |
| Bread intake 2nd quartile | 0.011 (0.139) | -0.029 (0.159) |
| Bread intake 3rd quartile | -0.134 (0.147) | -0.223 (0.168) |
| Bread intake 4th quartile | -0.195 (0.149) | -0.202 (0.170) |
| Sweet intake 2nd quartile | -0.093 (0.142) | 0.083 (0.165) |
| Sweet intake 3rd quartile | -0.236 (0.147) | -0.170 (0.173) |
| Sweet intake 4th quartile | -0.120 (0.146) | 0.010 (0.170) |
| Drink intake 2nd quartile | -0.116 (0.142) | -0.153 (0.164) |
| Drink intake 3rd quartile | -0.127 (0.146) | -0.068 (0.166) |
| Drink intake 4th quartile | -0.231 (0.146) | -0.285 (0.169) |
| Processed meat intake 2nd quartile | -0.154 (0.149) | -0.247 (0.169) |
| Processed meat intake 3rd quartile | 0.000 (0.149) | -0.057 (0.170) |
| Processed meat intake 4th quartile | -0.052 (0.162) | -0.174 (0.186) |
| Processed plant intake above median | 0.072 (0.119) | 0.187 (0.133) |
| Fruit intake 2nd quartile | 0.148 (0.154) | 0.118 (0.174) |
| Fruit intake 3rd quartile | 0.127 (0.155) | 0.085 (0.177) |
| Fruit intake 4th quartile | 0.133 (0.171) | -0.158 (0.197) |
| Vegetable intake 2nd quartile | 0.005 (0.152) | -0.011 (0.176) |
| Vegetable intake 3rd quartile | -0.001 (0.156) | -0.007 (0.178) |
| Vegetable intake 4th quartile | 0.090 (0.161) | 0.149 (0.182) |
| Red meat intake 2nd tertile | 0.033 (0.125) | -0.042 (0.145) |
| Red meat intake 3rd tertile | -0.082 (0.131) | -0.057 (0.148) |
| White meat intake 2nd quartile | -0.053 (0.148) | -0.108 (0.172) |
| White meat intake 3rd quartile | -0.064 (0.148) | -0.022 (0.167) |
| White meat intake 4th quartile | -0.006 (0.147) | -0.036 (0.169) |
| White fish intake 2nd quartile | -0.309 (0.157) | -0.335 (0.181) |
| White fish intake 3rd quartile | -0.125 (0.149) | -0.085 (0.170) |
| White fish intake 4th quartile | 0.151 (0.145) | 0.095 (0.167) |

**Table S6.6** Parameter estimates for risk factors for objective flare in Inflammatory Bowel disease, as inferred in univariate analyses (adjusting for known risk factors), in main and sensitivity analyses.

|  | **Main (SE)** | **Sensitivity (SE)** |
| --- | --- | --- |
| Meat protein 2nd quartile | 0.285 (0.203) | 0.289 (0.244) |
| Meat protein 3rd quartile | 0.312 (0.201) | 0.383 (0.234) |
| Meat protein 4th quartile | 0.408 (0.204) | 0.403 (0.239) |
| Overall meat intake 2nd quartile | -0.186 (0.205) | -0.257 (0.237) |
| Overall meat intake 3rd quartile | 0.190 (0.197) | 0.084 (0.228) |
| Overall meat intake 4th quartile | 0.263 (0.210) | 0.085 (0.243) |
| Overall fish intake 2nd quartile | -0.459 (0.204) | -0.419 (0.249) |
| Overall fish intake 3rd quartile | -0.302 (0.196) | 0.033 (0.223) |
| Overall fish intake 4th quartile | -0.029 (0.187) | -0.016 (0.231) |
| Dietary fibre 2nd quartile | 0.141 (0.200) | 0.004 (0.236) |
| Dietary fibre 3rd quartile | 0.338 (0.205) | 0.173 (0.243) |
| Dietary fibre 4th quartile | 0.244 (0.213) | 0.131 (0.246) |
| PUFA 2nd quartile | 0.047 (0.191) | -0.113 (0.231) |
| PUFA 3rd quartile | -0.120 (0.195) | -0.091 (0.227) |
| PUFA 4th quartile | 0.065 (0.191) | 0.151 (0.220) |
| NOVA Score 2 | 0.390 (0.194) | 0.388 (0.232) |
| NOVA Score 3 | 0.372 (0.200) | 0.287 (0.235) |
| NOVA Score 4 | 0.202 (0.204) | 0.071 (0.247) |
| %UPF 2nd quartile | -0.288 (0.184) | -0.384 (0.216) |
| %UPF 3rd quartile | -0.652 (0.202) | -0.762 (0.242) |
| %UPF 4th quartile | -0.209 (0.188) | -0.179 (0.215) |
| Bread intake 2nd quartile | 0.007 (0.184) | 0.012 (0.216) |
| Bread intake 3rd quartile | -0.055 (0.191) | -0.135 (0.223) |
| Bread intake 4th quartile | -0.403 (0.204) | -0.405 (0.238) |
| Sweet intake 2nd quartile | -0.349 (0.191) | -0.442 (0.223) |
| Sweet intake 3rd quartile | -0.473 (0.197) | -0.611 (0.230) |
| Sweet intake 4th quartile | -0.066 (0.186) | -0.265 (0.217) |
| Drink intake 2nd quartile | -0.173 (0.194) | -0.368 (0.232) |
| Drink intake 3rd quartile | -0.011 (0.191) | 0.070 (0.213) |
| Drink intake 4th quartile | -0.044 (0.194) | -0.305 (0.233) |
| Processed meat intake 2nd quartile | 0.083 (0.199) | 0.060 (0.234) |
| Processed meat intake 3rd quartile | 0.179 (0.200) | 0.181 (0.232) |
| Processed meat intake 4th quartile | 0.050 (0.221) | 0.058 (0.258) |
| Processed plant intake above median | -0.046 (0.162) | -0.075 (0.190) |
| Fruit intake 2nd quartile | -0.160 (0.204) | -0.261 (0.237) |
| Fruit intake 3rd quartile | -0.019 (0.201) | -0.037 (0.235) |
| Fruit intake 4th quartile | 0.011 (0.220) | -0.045 (0.252) |
| Vegetable intake 2nd quartile | 0.027 (0.203) | -0.150 (0.242) |
| Vegetable intake 3rd quartile | 0.181 (0.205) | 0.121 (0.234) |
| Vegetable intake 4th quartile | 0.337 (0.216) | 0.347 (0.244) |
| Red meat intake 2nd tertile | 0.004 (0.167) | 0.153 (0.198) |
| Red meat intake 3rd tertile | 0.112 (0.169) | 0.267 (0.195) |
| White meat intake 2nd quartile | -0.109 (0.199) | -0.228 (0.234) |
| White meat intake 3rd quartile | -0.101 (0.196) | -0.120 (0.225) |
| White meat intake 4th quartile | 0.189 (0.190) | 0.067 (0.222) |
| White fish intake 2nd quartile | -0.588 (0.204) | -0.513 (0.247) |
| White fish intake 3rd quartile | -0.451 (0.200) | -0.266 (0.234) |
| White fish intake 4th quartile | -0.094 (0.186) | 0.101 (0.223) |

**Table S7.1** P-values for risk factors for patient-reported flare in Crohn’s disease, as inferred in univariate analyses (adjusting for known risk factors), and adjusted p-values for those risk factors that were not specified in the SAP.

|  | **P-value** | **Adj P-value** |
| --- | --- | --- |
| Meat protein 2nd quartile | 0.99 | — |
| Meat protein 3rd quartile | 0.51 | — |
| Meat protein 4th quartile | 0.74 | — |
| Overall meat intake 2nd quartile | 0.90 | 1.00 |
| Overall meat intake 3rd quartile | 0.61 | 1.00 |
| Overall meat intake 4th quartile | 0.64 | 1.00 |
| Overall fish intake 2nd quartile | 0.022 | 1.00 |
| Overall fish intake 3rd quartile | 0.082 | 1.00 |
| Overall fish intake 4th quartile | 0.50 | 1.00 |
| Dietary fibre 2nd quartile | 0.19 | — |
| Dietary fibre 3rd quartile | 0.62 | — |
| Dietary fibre 4th quartile | 0.86 | — |
| PUFA 2nd quartile | 0.18 | — |
| PUFA 3rd quartile | 0.052 | — |
| PUFA 4th quartile | 0.74 | — |
| NOVA Score 2 | 0.78 | 1.00 |
| NOVA Score 3 | 0.60 | 1.00 |
| NOVA Score 4 | 0.85 | 1.00 |
| %UPF 2nd quartile | 0.62 | — |
| %UPF 3rd quartile | 0.51 | — |
| %UPF 4th quartile | 0.91 | — |
| Bread intake 2nd quartile | 0.082 | 1.00 |
| Bread intake 3rd quartile | 0.21 | 1.00 |
| Bread intake 4th quartile | 0.39 | 1.00 |
| Sweet intake 2nd quartile | 0.78 | 1.00 |
| Sweet intake 3rd quartile | 0.34 | 1.00 |
| Sweet intake 4th quartile | 0.88 | 1.00 |
| Drink intake 2nd quartile | 0.98 | 1.00 |
| Drink intake 3rd quartile | 0.67 | 1.00 |
| Drink intake 4th quartile | 0.21 | 1.00 |
| Processed meat intake 2nd quartile | 0.90 | 1.00 |
| Processed meat intake 3rd quartile | 0.30 | 1.00 |
| Processed meat intake 4th quartile | 0.54 | 1.00 |
| Processed plant intake above median | 0.41 | 1.00 |
| Fruit intake 2nd quartile | 0.60 | 1.00 |
| Fruit intake 3rd quartile | 0.81 | 1.00 |
| Fruit intake 4th quartile | 0.85 | 1.00 |
| Vegetable intake 2nd quartile | 0.94 | 1.00 |
| Vegetable intake 3rd quartile | 0.34 | 1.00 |
| Vegetable intake 4th quartile | 0.98 | 1.00 |
| Red meat intake 2nd tertile | 0.37 | 1.00 |
| Red meat intake 3rd tertile | 0.64 | 1.00 |
| White meat intake 2nd quartile | 0.42 | 1.00 |
| White meat intake 3rd quartile | 0.26 | 1.00 |
| White meat intake 4th quartile | 0.75 | 1.00 |
| White fish intake 2nd quartile | 0.001 | 0.32 |
| White fish intake 3rd quartile | 0.051 | 1.00 |
| White fish intake 4th quartile | 0.29 | 1.00 |

**Table S7.2** P-values for risk factors for objective flare in Crohn’s disease, as inferred in univariate analyses (adjusting for known risk factors), and adjusted p-values for those risk factors that were not specified in the SAP.

|  | **P-value** | **Adj P-value** |
| --- | --- | --- |
| Meat protein 2nd quartile | 0.35 | — |
| Meat protein 3rd quartile | 0.54 | — |
| Meat protein 4th quartile | 0.91 | — |
| Overall meat intake 2nd quartile | 0.63 | 1.00 |
| Overall meat intake 3rd quartile | 0.73 | 1.00 |
| Overall meat intake 4th quartile | 0.60 | 1.00 |
| Overall fish intake 2nd quartile | 0.008 | 1.00 |
| Overall fish intake 3rd quartile | 0.068 | 1.00 |
| Overall fish intake 4th quartile | 0.33 | 1.00 |
| Dietary fibre 2nd quartile | 0.93 | — |
| Dietary fibre 3rd quartile | 0.21 | — |
| Dietary fibre 4th quartile | 0.29 | — |
| PUFA 2nd quartile | 0.73 | — |
| PUFA 3rd quartile | 0.75 | — |
| PUFA 4th quartile | 0.35 | — |
| NOVA Score 2 | 0.15 | 1.00 |
| NOVA Score 3 | 0.28 | 1.00 |
| NOVA Score 4 | 0.88 | 1.00 |
| %UPF 2nd quartile | 0.95 | — |
| %UPF 3rd quartile | 0.10 | — |
| %UPF 4th quartile | 0.59 | — |
| Bread intake 2nd quartile | 0.16 | 1.00 |
| Bread intake 3rd quartile | 0.19 | 1.00 |
| Bread intake 4th quartile | 0.088 | 1.00 |
| Sweet intake 2nd quartile | 0.30 | 1.00 |
| Sweet intake 3rd quartile | 0.35 | 1.00 |
| Sweet intake 4th quartile | 0.81 | 1.00 |
| Drink intake 2nd quartile | 0.78 | 1.00 |
| Drink intake 3rd quartile | 0.29 | 1.00 |
| Drink intake 4th quartile | 0.20 | 1.00 |
| Processed meat intake 2nd quartile | 0.90 | 1.00 |
| Processed meat intake 3rd quartile | 0.45 | 1.00 |
| Processed meat intake 4th quartile | 0.65 | 1.00 |
| Processed plant intake above median | 0.55 | 1.00 |
| Fruit intake 2nd quartile | 0.26 | 1.00 |
| Fruit intake 3rd quartile | 0.97 | 1.00 |
| Fruit intake 4th quartile | 0.64 | 1.00 |
| Vegetable intake 2nd quartile | 0.77 | 1.00 |
| Vegetable intake 3rd quartile | 0.66 | 1.00 |
| Vegetable intake 4th quartile | 0.20 | 1.00 |
| Red meat intake 2nd tertile | 0.47 | 1.00 |
| Red meat intake 3rd tertile | 0.10 | 1.00 |
| White meat intake 2nd quartile | 0.030 | 1.00 |
| White meat intake 3rd quartile | 0.023 | 1.00 |
| White meat intake 4th quartile | 0.24 | 1.00 |
| **White fish intake 2nd quartile** | **<0.001** | **0.044** |
| White fish intake 3rd quartile | 0.027 | 1.00 |
| White fish intake 4th quartile | 0.30 | 1.00 |

**Table S7.3** P-values for risk factors for patient-reported flare in Ulcerative colitis, as inferred in univariate analyses (adjusting for known risk factors), and adjusted p-values for those risk factors that were not specified in the SAP.

|  | **P-value** | **Adj P-value** |
| --- | --- | --- |
| Meat protein 2nd quartile | 0.43 | — |
| Meat protein 3rd quartile | 0.13 | — |
| Meat protein 4th quartile | 0.26 | — |
| Overall meat intake 2nd quartile | 0.70 | 1.00 |
| Overall meat intake 3rd quartile | 0.31 | 1.00 |
| Overall meat intake 4th quartile | 0.75 | 1.00 |
| Overall fish intake 2nd quartile | 0.41 | 1.00 |
| Overall fish intake 3rd quartile | 0.52 | 1.00 |
| Overall fish intake 4th quartile | 0.047 | 1.00 |
| Dietary fibre 2nd quartile | 0.51 | — |
| Dietary fibre 3rd quartile | 0.88 | — |
| Dietary fibre 4th quartile | 0.39 | — |
| PUFA 2nd quartile | 0.57 | — |
| PUFA 3rd quartile | 0.47 | — |
| PUFA 4th quartile | 0.40 | — |
| NOVA Score 2 | 0.50 | 1.00 |
| NOVA Score 3 | 0.58 | 1.00 |
| NOVA Score 4 | 0.46 | 1.00 |
| **%UPF 2nd quartile** | **0.006** | **—** |
| **%UPF 3rd quartile** | **0.004** | **—** |
| **%UPF 4th quartile** | **0.010** | **—** |
| Bread intake 2nd quartile | 0.076 | 1.00 |
| Bread intake 3rd quartile | 0.98 | 1.00 |
| Bread intake 4th quartile | 0.24 | 1.00 |
| Sweet intake 2nd quartile | 0.22 | 1.00 |
| Sweet intake 3rd quartile | 0.16 | 1.00 |
| Sweet intake 4th quartile | 0.12 | 1.00 |
| Drink intake 2nd quartile | 0.24 | 1.00 |
| Drink intake 3rd quartile | 0.64 | 1.00 |
| Drink intake 4th quartile | 0.46 | 1.00 |
| Processed meat intake 2nd quartile | 0.11 | 1.00 |
| Processed meat intake 3rd quartile | 0.32 | 1.00 |
| Processed meat intake 4th quartile | 0.30 | 1.00 |
| Processed plant intake above median | 0.68 | 1.00 |
| Fruit intake 2nd quartile | 0.69 | 1.00 |
| Fruit intake 3rd quartile | 0.52 | 1.00 |
| Fruit intake 4th quartile | 0.70 | 1.00 |
| Vegetable intake 2nd quartile | 0.67 | 1.00 |
| Vegetable intake 3rd quartile | 0.26 | 1.00 |
| Vegetable intake 4th quartile | 0.34 | 1.00 |
| Red meat intake 2nd tertile | 0.28 | 1.00 |
| Red meat intake 3rd tertile | 0.63 | 1.00 |
| White meat intake 2nd quartile | 0.70 | 1.00 |
| White meat intake 3rd quartile | 0.80 | 1.00 |
| White meat intake 4th quartile | 0.90 | 1.00 |
| White fish intake 2nd quartile | 0.57 | 1.00 |
| White fish intake 3rd quartile | 0.63 | 1.00 |
| White fish intake 4th quartile | 0.021 | 1.00 |

**Table S7.4** P-values for risk factors for objective flare in Ulcerative colitis, as inferred in univariate analyses (adjusting for known risk factors), and adjusted p-values for those risk factors that were not specified in the SAP.

|  | **P-value** | **Adj P-value** |
| --- | --- | --- |
| Meat protein 2nd quartile | 0.26 | — |
| Meat protein 3rd quartile | 0.13 | — |
| Meat protein 4th quartile | 0.007 | — |
| Overall meat intake 2nd quartile | 0.39 | 1.00 |
| Overall meat intake 3rd quartile | 0.13 | 1.00 |
| Overall meat intake 4th quartile | 0.030 | 1.00 |
| Overall fish intake 2nd quartile | 0.43 | 1.00 |
| Overall fish intake 3rd quartile | 0.61 | 1.00 |
| Overall fish intake 4th quartile | 0.57 | 1.00 |
| Dietary fibre 2nd quartile | 0.44 | — |
| Dietary fibre 3rd quartile | 0.23 | — |
| Dietary fibre 4th quartile | 0.61 | — |
| PUFA 2nd quartile | 0.73 | — |
| PUFA 3rd quartile | 0.31 | — |
| PUFA 4th quartile | 0.59 | — |
| NOVA Score 2 | 0.12 | 1.00 |
| NOVA Score 3 | 0.089 | 1.00 |
| NOVA Score 4 | 0.20 | 1.00 |
| **%UPF 2nd quartile** | **0.024** | **—** |
| **%UPF 3rd quartile** | **0.005** | **—** |
| %UPF 4th quartile | 0.32 | — |
| Bread intake 2nd quartile | 0.30 | 1.00 |
| Bread intake 3rd quartile | 0.66 | 1.00 |
| Bread intake 4th quartile | 0.18 | 1.00 |
| Sweet intake 2nd quartile | 0.16 | 1.00 |
| Sweet intake 3rd quartile | 0.018 | 1.00 |
| Sweet intake 4th quartile | 0.65 | 1.00 |
| Drink intake 2nd quartile | 0.32 | 1.00 |
| Drink intake 3rd quartile | 0.27 | 1.00 |
| Drink intake 4th quartile | 0.36 | 1.00 |
| Processed meat intake 2nd quartile | 0.53 | 1.00 |
| Processed meat intake 3rd quartile | 0.68 | 1.00 |
| Processed meat intake 4th quartile | 0.38 | 1.00 |
| Processed plant intake above median | 0.31 | 1.00 |
| Fruit intake 2nd quartile | 0.89 | 1.00 |
| Fruit intake 3rd quartile | 0.69 | 1.00 |
| Fruit intake 4th quartile | 0.71 | 1.00 |
| Vegetable intake 2nd quartile | 0.43 | 1.00 |
| Vegetable intake 3rd quartile | 0.036 | 1.00 |
| Vegetable intake 4th quartile | 0.20 | 1.00 |
| Red meat intake 2nd tertile | 0.26 | 1.00 |
| Red meat intake 3rd tertile | 0.010 | 1.00 |
| White meat intake 2nd quartile | 0.23 | 1.00 |
| White meat intake 3rd quartile | 0.24 | 1.00 |
| White meat intake 4th quartile | 0.021 | 1.00 |
| White fish intake 2nd quartile | 0.76 | 1.00 |
| White fish intake 3rd quartile | 0.27 | 1.00 |
| White fish intake 4th quartile | 0.87 | 1.00 |

**Table S7.5** P-values for risk factors for patient-reported flare in Inflammatory Bowel disease, as inferred in univariate analyses (adjusting for known risk factors), and adjusted p-values for those risk factors that were not specified in the SAP.

|  | **P-value** | **Adj P-value** |
| --- | --- | --- |
| Meat protein 2nd quartile | 0.58 | — |
| Meat protein 3rd quartile | 0.47 | — |
| Meat protein 4th quartile | 0.53 | — |
| Overall meat intake 2nd quartile | 0.81 | 1.00 |
| Overall meat intake 3rd quartile | 0.59 | 1.00 |
| Overall meat intake 4th quartile | 0.98 | 1.00 |
| Overall fish intake 2nd quartile | 0.33 | 1.00 |
| Overall fish intake 3rd quartile | 0.66 | 1.00 |
| Overall fish intake 4th quartile | 0.28 | 1.00 |
| Dietary fibre 2nd quartile | 0.24 | — |
| Dietary fibre 3rd quartile | 0.70 | — |
| Dietary fibre 4th quartile | 0.49 | — |
| PUFA 2nd quartile | 0.28 | — |
| PUFA 3rd quartile | 0.072 | — |
| PUFA 4th quartile | 0.73 | — |
| NOVA Score 2 | 0.43 | 1.00 |
| NOVA Score 3 | 0.91 | 1.00 |
| NOVA Score 4 | 0.57 | 1.00 |
| %UPF 2nd quartile | 0.17 | — |
| %UPF 3rd quartile | 0.13 | — |
| **%UPF 4th quartile** | **0.046** | **—** |
| Bread intake 2nd quartile | 0.94 | 1.00 |
| Bread intake 3rd quartile | 0.36 | 1.00 |
| Bread intake 4th quartile | 0.19 | 1.00 |
| Sweet intake 2nd quartile | 0.51 | 1.00 |
| Sweet intake 3rd quartile | 0.11 | 1.00 |
| Sweet intake 4th quartile | 0.41 | 1.00 |
| Drink intake 2nd quartile | 0.41 | 1.00 |
| Drink intake 3rd quartile | 0.38 | 1.00 |
| Drink intake 4th quartile | 0.11 | 1.00 |
| Processed meat intake 2nd quartile | 0.30 | 1.00 |
| Processed meat intake 3rd quartile | 1.00 | 1.00 |
| Processed meat intake 4th quartile | 0.75 | 1.00 |
| Processed plant intake above median | 0.54 | 1.00 |
| Fruit intake 2nd quartile | 0.34 | 1.00 |
| Fruit intake 3rd quartile | 0.41 | 1.00 |
| Fruit intake 4th quartile | 0.44 | 1.00 |
| Vegetable intake 2nd quartile | 0.97 | 1.00 |
| Vegetable intake 3rd quartile | 1.00 | 1.00 |
| Vegetable intake 4th quartile | 0.57 | 1.00 |
| Red meat intake 2nd tertile | 0.79 | 1.00 |
| Red meat intake 3rd tertile | 0.53 | 1.00 |
| White meat intake 2nd quartile | 0.72 | 1.00 |
| White meat intake 3rd quartile | 0.67 | 1.00 |
| White meat intake 4th quartile | 0.97 | 1.00 |
| White fish intake 2nd quartile | 0.049 | 1.00 |
| White fish intake 3rd quartile | 0.40 | 1.00 |
| White fish intake 4th quartile | 0.30 | 1.00 |

**Table S7.6** P-values for risk factors for objective flare in Inflammatory Bowel disease, as inferred in univariate analyses (adjusting for known risk factors), and adjusted p-values for those risk factors that were not specified in the SAP.

|  | **P-value** | **Adj P-value** |
| --- | --- | --- |
| Meat protein 2nd quartile | 0.16 | — |
| Meat protein 3rd quartile | 0.12 | — |
| **Meat protein 4th quartile** | **0.045** | **—** |
| Overall meat intake 2nd quartile | 0.36 | 1.00 |
| Overall meat intake 3rd quartile | 0.34 | 1.00 |
| Overall meat intake 4th quartile | 0.21 | 1.00 |
| Overall fish intake 2nd quartile | 0.025 | 1.00 |
| Overall fish intake 3rd quartile | 0.12 | 1.00 |
| Overall fish intake 4th quartile | 0.88 | 1.00 |
| Dietary fibre 2nd quartile | 0.48 | — |
| Dietary fibre 3rd quartile | 0.100 | — |
| Dietary fibre 4th quartile | 0.25 | — |
| PUFA 2nd quartile | 0.81 | — |
| PUFA 3rd quartile | 0.54 | — |
| PUFA 4th quartile | 0.73 | — |
| NOVA Score 2 | 0.045 | 1.00 |
| NOVA Score 3 | 0.063 | 1.00 |
| NOVA Score 4 | 0.32 | 1.00 |
| %UPF 2nd quartile | 0.12 | — |
| **%UPF 3rd quartile** | **0.001** | **—** |
| %UPF 4th quartile | 0.27 | — |
| Bread intake 2nd quartile | 0.97 | 1.00 |
| Bread intake 3rd quartile | 0.77 | 1.00 |
| Bread intake 4th quartile | 0.048 | 1.00 |
| Sweet intake 2nd quartile | 0.068 | 1.00 |
| Sweet intake 3rd quartile | 0.016 | 1.00 |
| Sweet intake 4th quartile | 0.72 | 1.00 |
| Drink intake 2nd quartile | 0.37 | 1.00 |
| Drink intake 3rd quartile | 0.95 | 1.00 |
| Drink intake 4th quartile | 0.82 | 1.00 |
| Processed meat intake 2nd quartile | 0.68 | 1.00 |
| Processed meat intake 3rd quartile | 0.37 | 1.00 |
| Processed meat intake 4th quartile | 0.82 | 1.00 |
| Processed plant intake above median | 0.78 | 1.00 |
| Fruit intake 2nd quartile | 0.43 | 1.00 |
| Fruit intake 3rd quartile | 0.93 | 1.00 |
| Fruit intake 4th quartile | 0.96 | 1.00 |
| Vegetable intake 2nd quartile | 0.89 | 1.00 |
| Vegetable intake 3rd quartile | 0.38 | 1.00 |
| Vegetable intake 4th quartile | 0.12 | 1.00 |
| Red meat intake 2nd tertile | 0.98 | 1.00 |
| Red meat intake 3rd tertile | 0.51 | 1.00 |
| White meat intake 2nd quartile | 0.58 | 1.00 |
| White meat intake 3rd quartile | 0.61 | 1.00 |
| White meat intake 4th quartile | 0.32 | 1.00 |
| White fish intake 2nd quartile | 0.004 | 0.86 |
| White fish intake 3rd quartile | 0.024 | 1.00 |
| White fish intake 4th quartile | 0.61 | 1.00 |
